# Iron overload reduces synthesis and elimination of bile acids in rat liver

**DOI:** 10.1038/s41598-019-46150-7

**Published:** 2019-07-05

**Authors:** Alena Prasnicka, Hana Lastuvkova, Fatemeh Alaei Faradonbeh, Jolana Cermanova, Milos Hroch, Jaroslav Mokry, Eva Dolezelova, Petr Pavek, Katerina Zizalova, Libor Vitek, Petr Nachtigal, Stanislav Micuda

**Affiliations:** 1 Department of Pharmacology, Charles University, Faculty of Medicine in Hradec Kralove, Hradec Kralove, Czech Republic; 2 Department of Medical Biochemistry, Charles University, Faculty of Medicine in Hradec Kralove, Hradec Kralove, Czech Republic; 3 Department of Histology and Embryology, Charles University, Faculty of Medicine in Hradec Kralove, Hradec Kralove, Czech Republic; 4 Department of Biological and Medical Sciences, Charles University, Faculty of Pharmacy in Hradec Kralove, Hradec Kralove, Czech Republic; 5 Department of Pharmacology and Toxicology, Charles University, Faculty of Pharmacy in Hradec Kralove, Hradec Kralove, Czech Republic; 6 Department of Medical Biochemistry and Laboratory Diagnostics, 1st Faculty of Medicine, Charles University, Prague, Czech Republic

**Keywords:** Hepatotoxicity, Experimental models of disease

## Abstract

Excessive iron accumulation in the liver, which accompanies certain genetic or metabolic diseases, impairs bile acids (BA) synthesis, but the influence of iron on the complex process of BA homeostasis is unknown. Thus, we evaluated the effect of iron overload (IO) on BA turnover in rats. Compared with control rats, IO (8 intraperitoneal doses of 100 mg/kg every other day) significantly decreased bile flow as a consequence of decreased biliary BA secretion. This decrease was associated with reduced expression of Cyp7a1, the rate limiting enzyme in the conversion of cholesterol to BA, and decreased expression of Bsep, the transporter responsible for BA efflux into bile. However, IO did not change net BA content in faeces in response to increased intestinal conversion of BA into hyodeoxycholic acid. In addition, IO increased plasma cholesterol concentrations, which corresponded with reduced Cyp7a1 expression and increased expression of Hmgcr, the rate-limiting enzyme in *de novo* cholesterol synthesis. In summary, this study describes the mechanisms impairing synthesis, biliary secretion and intestinal processing of BA during IO. Altered elimination pathways for BA and cholesterol may interfere with the pathophysiology of liver damage accompanying liver diseases with excessive iron deposition.

## Introduction

Bile production is an essential function of the liver and serves as an irreplaceable excretory pathway for elimination of lipophilic endo- and xenobiotics such as cholesterol, BA, bilirubin or drugs^1^. Moreover, as major components of bile, BA are required for micelle formation, intestinal fat digestion, regulation of bacterial growth, and immune response and production of regulatory mediators released to portal circulation such as fibroblast growth factor 19 or glucagon-like peptide 1. In addition, BA as the major metabolites of cholesterol, act as hormones by agonism at several receptors such as farnesoid X receptor (FXR), the G protein-coupled bile acid receptor 1 (TGR5), sphingosine-1-phosphate receptor 2, or pregnane X receptor (PXR), and regulate numerous liver functions including glucose and triglyceride metabolism^2^. Stimulation of these receptors demonstrates promising positive effects in liver diseases such as nonalcoholic steatohepatitis (NASH) or intrahepatic cholestasis^3^. On the other hand, BA accumulated during different forms of cholestasis may have a direct toxic effect on liver cells and tissues. Regulation of bile production and BA homeostasis are therefore key events in liver physiology and pathophysiology.

Iron is an essential trace element, in particular required to form haem for synthesis of haemoglobin, myoglobin or P450 enzymes. As a highly reactive molecule, iron is also involved in the cellular redox balance and generation of hydroxyl radicals which are necessary for regulation of several intracellular events including response to stressors or mitochondrial dysfunction^4^. Excessive concentration of iron in cells induces oxidative stress with peroxidative decomposition of polyunsaturated fatty acids in membrane phospholipids, thereby altering vital organelle integrity and cell function^5,6^. The metabolism of iron is therefore tightly regulated to prevent tissue damage. However, IO can occur in subjects with genetic disorders such as hereditary haemochromatosis and beta thalassaemia, or secondary to IO during blood transfusion and haemolysis^7,8^. Iron toxicity occurs especially in the liver, where the iron is mainly stored, leading to ongoing damage and finally to cirrhosis. Moreover, increased liver iron stores accompany common metabolic pathologies such as insulin resistance, type 2 diabetes mellitus, metabolic syndrome, and nonalcoholic fatty liver disease (NAFLD)^9^. It is of note, that these metabolic disorders produce marked changes in BA metabolism and could be treated by agonists of FXR receptor, which is the most important BA sensor^10^. However, the relationship between IO and BA liver homeostatic pathways has not been studied in depth.

Indeed, plasma concentrations of BA during IO have not been yet measured and only limited evidence suggests that biliary BA excretion may be reduced by dietary IO^5^. This observation may be related to IO-mediated reduction in the expression of cholesterol 7α-hydroxylase (Cyp7a1), the rate-limiting enzyme for conversion of cholesterol to BA in rats^5,11,12^, although no association between liver iron concentration and Cyp7a1 was seen in this model of IO^13^. Reduction in Cyp7a1 may also explain increased serum cholesterol levels in iron-administered rats^5,14–17^. However, the effect of liver iron accumulation on other BA synthetic pathways and on biliary and faecal BA excretion is not known. Therefore, we postulated the hypothesis that iron accumulation in an organism markedly reduces elimination of BA.

In the present study we evaluated the effect of IO on the mechanisms responsible for BA homeostasis in rat liver and ileum. We showed that increased iron deposition in rat liver results in decreased bile formation due to reduced biliary BA secretion through downregulated Bsep and Mrp2 apical transporters. Plasma concentrations of BA were not significantly affected by IO because reduced biliary BA secretion was accompanied by reduced liver BA synthesis, intestinal BA processing, and increased basolateral output from hepatocytes and reduced uptake to hepatocytes.

## Results

### IO causes massive iron deposition in rat livers

**Figure 1:**
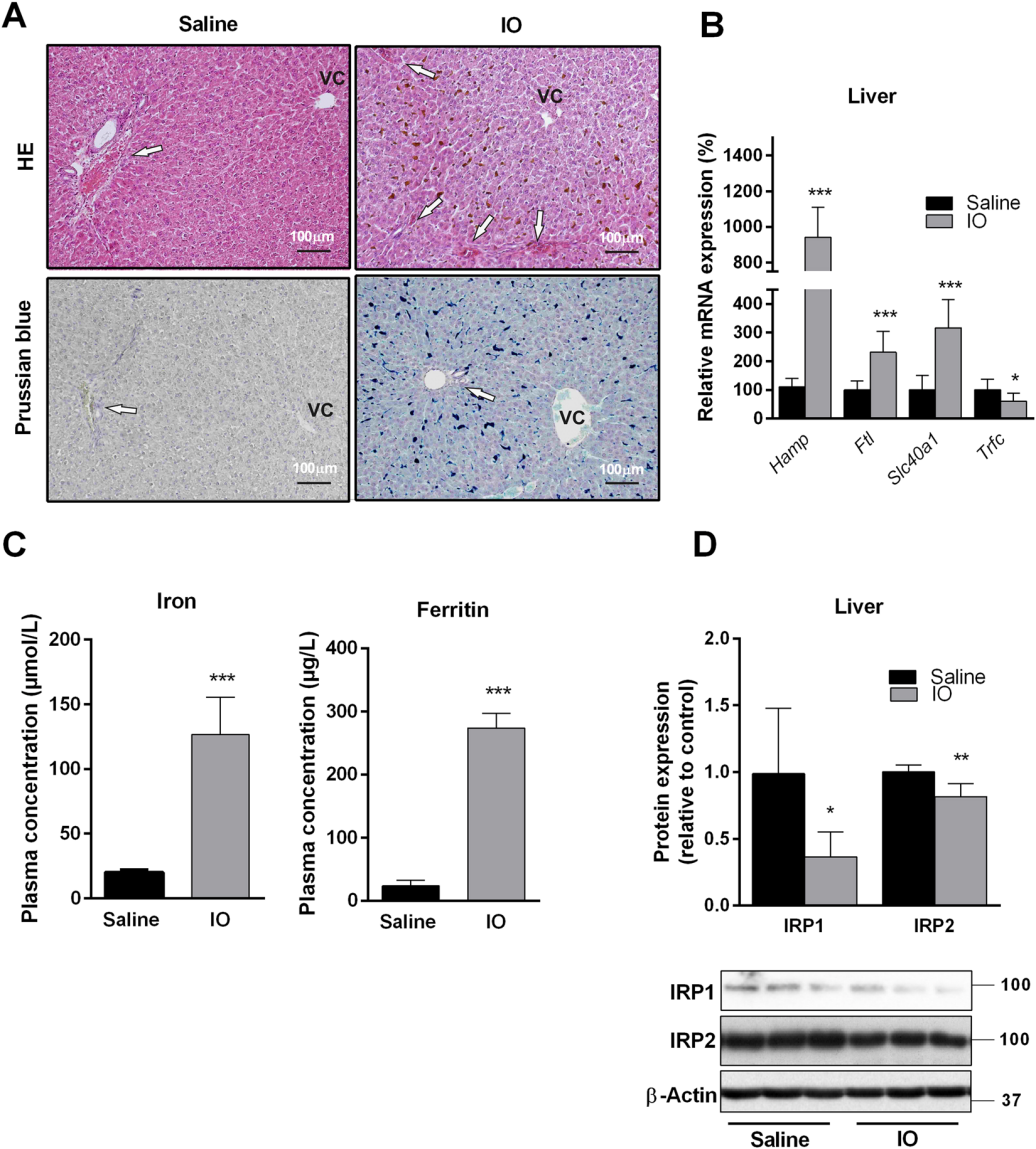
Excessive concentration of iron in IO rats administrated i.p. with 8 doses of gleptoferron every 2^nd^ day. (**A**) Representative liver histology, stained with haematoxylin-eosin staining (HE) and Prussian blue. Arrows indicate periportal areas at the periphery of classical liver lobule; VC – vena centralis. Scale bar 100 μm. (**B**) mRNA liver expression of hepcidin (*Hamp*), ferritin (*Ftl*), ferroportin (*Slc40a1*) and transferrin receptor (*Trfc*) determined by real-time RT-PCR. (**C**) Concentration of iron and ferritin in plasma. (**D**) Liver protein content of IRP1 and IRP2 (iron-responsive element-binding protein 1 and 2) normalized to β-actin. Values are mean ± SD (n = 6 in each group). *p < 0.05, **p < 0.01, ***p < 0.001 iron-treated vs. saline-treated rats.

To establish IO with significant iron liver accumulation in parenchymal and nonparenchymal cells, we used a validated rat model based on intraperitoneal (i.p.) administration of 8 doses (100 mg/kg per dose) of iron dextran-heptonic acid complex applied every other day^18^. This regimen resulted in massive iron liver deposition without significant hallmarks of hepatocellular or cholestatic injury as apparent from histological examination of haematoxylin-eosin (HE) and Prussian blue iron (PB) staining of liver sections (Fig. 1A). Massive iron deposition was apparent, especially in periportal zones of the liver acinus when compared with regions around the central vein as visualized by opalescent structures in HE staining and blue deposits in PB staining. This corresponds with previously reported data^8,19^. No apparent staining was present in the saline-administered animals. Liver weights were not changed by IO and were 13.5 ± 0.5 g in the saline-treated rats, and 14.2 ± 0.5 g in the IO rats. IO with excessive liver accumulation was further confirmed by increased plasma concentrations of iron and ferritin (Fig. 1C) and by increased liver mRNA expression of key iron metabolism associated genes such as hepcidin (*Hamp*), ferritin (*Ftl*), ferroportin (*Slc40a1*) and down regulation of transferrin receptor 1 (*Trfc*) (Fig. 1B). We also detected significantly reduced levels of iron-responsive element-binding protein 1 and iron-responsive element-binding protein 2 (IRP1 and IRP2) proteins (Fig. 1D), a markers of iron excess in the liver^20^. IRP1 and IRP2 were recently demonstrated as a positive regulator of *Cyp7a1* transcription^11^. These results demonstrated typical histological, biochemical and molecular hallmarks of significant iron deposition in the liver of treated animals.

### IO causes mild liver injury and oxidative stress

**Figure 2:**
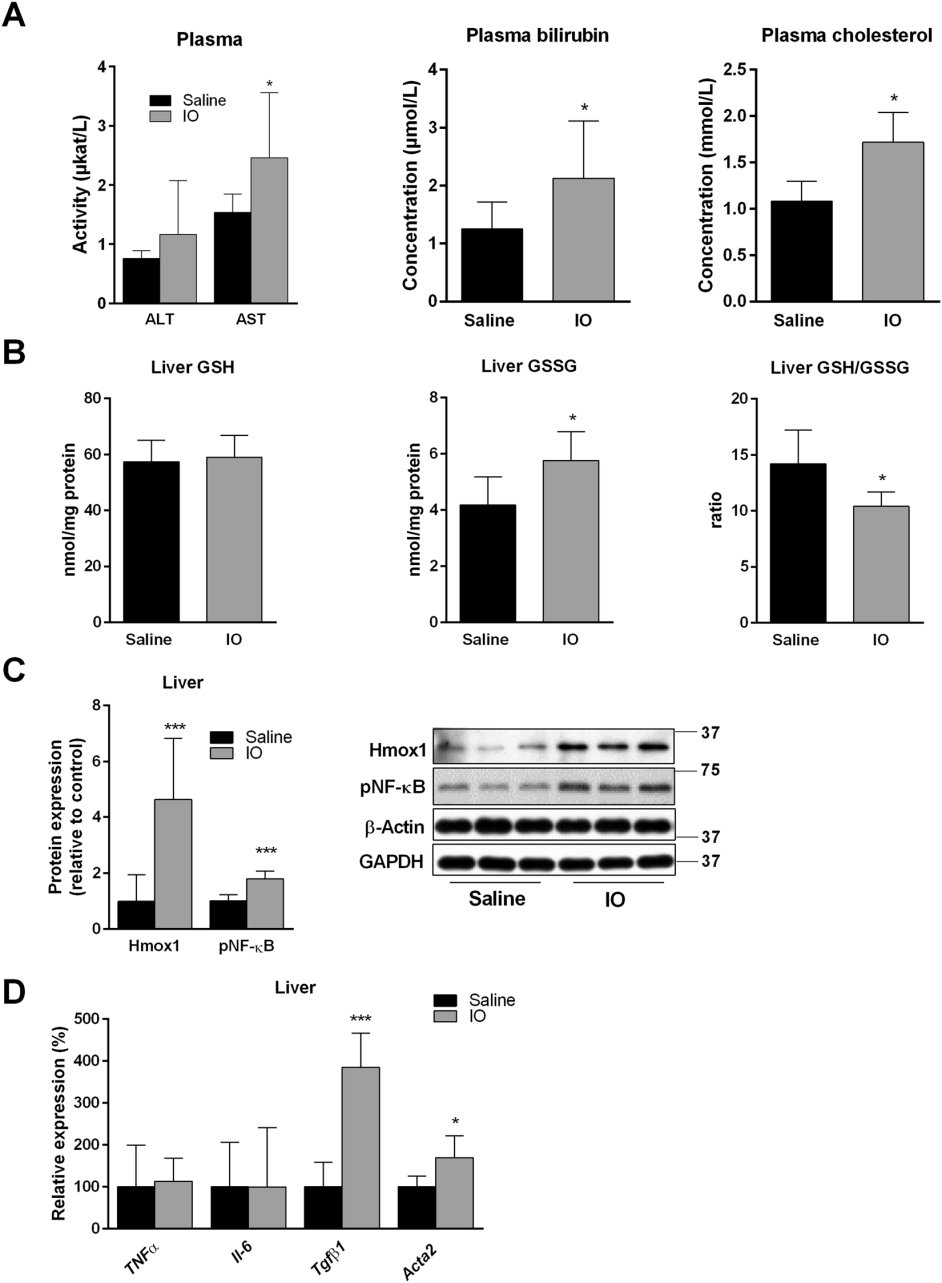
IO induced mild liver injury by activation of oxidative stress. (**A**) Activity and concentrations of ALT, AST, bilirubin, cholesterol in plasma. (**B**) Content of reduced and oxidized glutathione (GSSG) and GSH/GSSG ratio, determined by HPLC measurement. (**C**) Liver protein content of haem oxygenase (Hmox1) and phosphorylated NF-κB (p65) normalized to average of β-actin and Gapdh. (**D**) mRNA liver expressions of proinflammatory markers *TNFα*, *Il-6, Tgfβ1* and *Acta2* (encoding α-SMA protein) determined by real-time RT-PCR. Values are mean ± SD (n = 6 in each group). *p < 0.05, **p < 0.01, ***p < 0.001 iron-treated vs. saline-treated rats.

Excessive iron deposition in the liver can induce oxidative liver injury^6^. To determine liver injury in our IO model, we analysed the plasma and livers for corresponding biomarkers. The harmful effect of IO on liver functions was demonstrated by a mild but significant increase in aspartate transaminase (AST) activity, and plasma cholesterol and bilirubin concentrations (Fig. 2A). Induction of oxidative stress in the liver of IO rats was confirmed by the increased presence of glutathione in its oxidized (GSSG) form (Fig. 2B), reduced GSH/GSSG ratio (Fig. 2B) and by increased protein expression of stress-response molecules, haem oxygenase 1 (Hmox1) and phosphorylated NF-κB p65 subunit (Fig. 2C). These changes did not trigger acute inflammatory response in the liver, as suggested by the absence of inflammatory cell accumulation in histology sections (Fig. 1A) and unchanged gene expression of tumour necrosis factor (*Tnfα*) and interleukin 6 (*Il6*) (Fig. 2D). On the other hand, deposited iron activated *Tgfβ*1 production accompanied by increased expression of *Acta2 (*encoding α-SMA protein) (Fig. 2D), a marker of activated hepatic stellate cells, which was also recently demonstrated^21^.

### Bile flow is reduced by IO in response to reduced biliary secretion of BA

**Figure 3:**
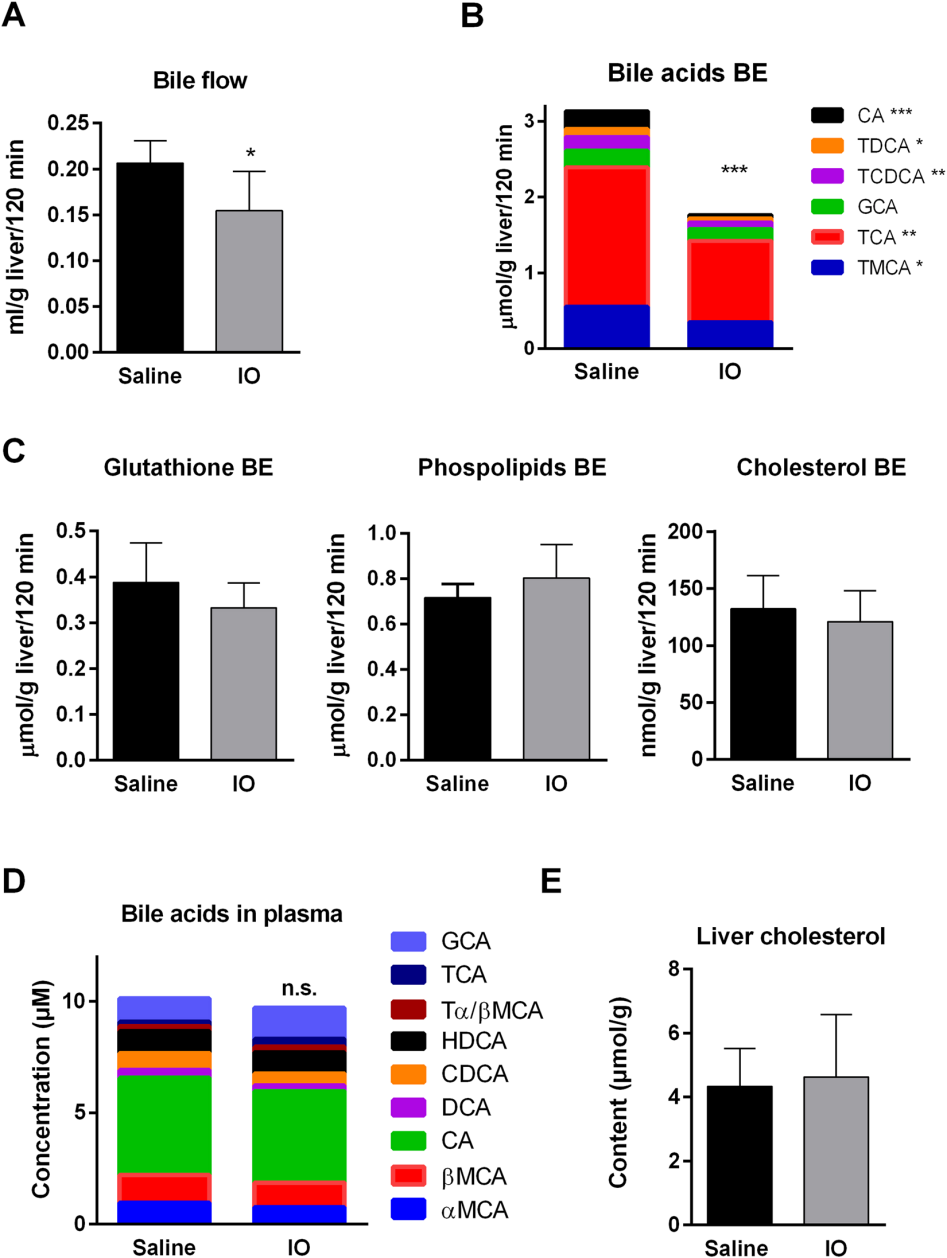
Bile flow was reduced in rats by IO in response to reduced biliary secretion of BA. (**A**) Rat bile duct was cannulated and bile was collected for 120 min. Then the bile flow was calculated from amount of collected bile to liver weight. (**B**,**D**) The concentrations of total and individual BAs in bile and plasma were measured by LC-MS analysis. (**C**,**E**) Biliary secretions of phospholipids, glutathione, cholesterol and hepatic content of cholesterol were determined by available commercial kits. Values are mean ± SD (n = 6 in each group). *p < 0.05, **p < 0.01, ***p < 0.001 iron-treated vs. saline-treated rats.

Reduced biliary secretion of total BA during IO was reported previously in a single study^5^. To elaborate this finding, we performed a bile collection study with analysis of BA spectra and other major components of bile including BA-independent flow based mainly on biliary secretion of glutathione. IO caused significant reduction of net bile flow in rats (Fig. 3A), accompanied by decreased biliary secretion of BA. Individual BA were proportionally reduced in the presence of IO (Fig. 3B). Changes in biliary secretion of individual bile acids presents Supplementary Table 3. Biliary secretion of glutathione, cholesterol and phospholipids did not significantly differ between the control and IO rats (Fig. 3C). Similarly, concentrations of BA and their spectra in plasma and the concentration of cholesterol in liver were not changed by iron administration (Fig. 3D,E, Supplementary Table 3). Our data indicate that the reduction of bile production by IO reflected reduced biliary secretion of BA.

### Impact of IO on liver expression of molecules responsible for BA and cholesterol turnover

**Figure 4:**
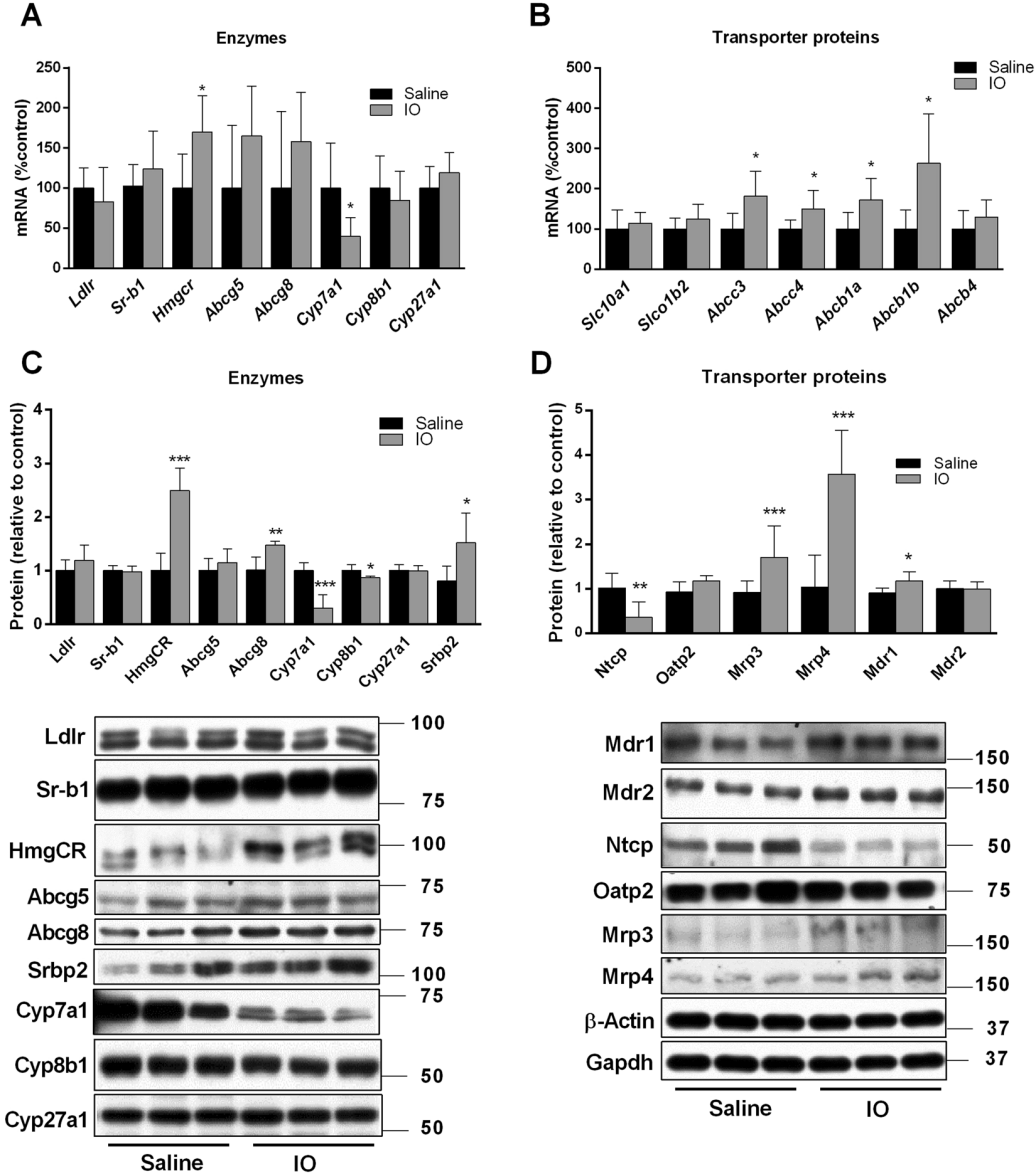
Impact of IO on liver mRNA and protein expression responsible for BA and cholesterol turnover. Isolated mRNAs (**A**,**B**), and proteins (**C**,**D**) from liver tissue were used to determine the expression of transporters and enzymes responsible for cholesterol and BAs homeostasis. The expression of these genes was analysed by a real-time RT-PCR system and proteins by western blot immunodetection. Values are mean ± SD (n = 6 in each group). *p < 0.05, **p < 0.01, ***p < 0.001 iron-treated vs. saline-treated rats.

**Figure 5:**
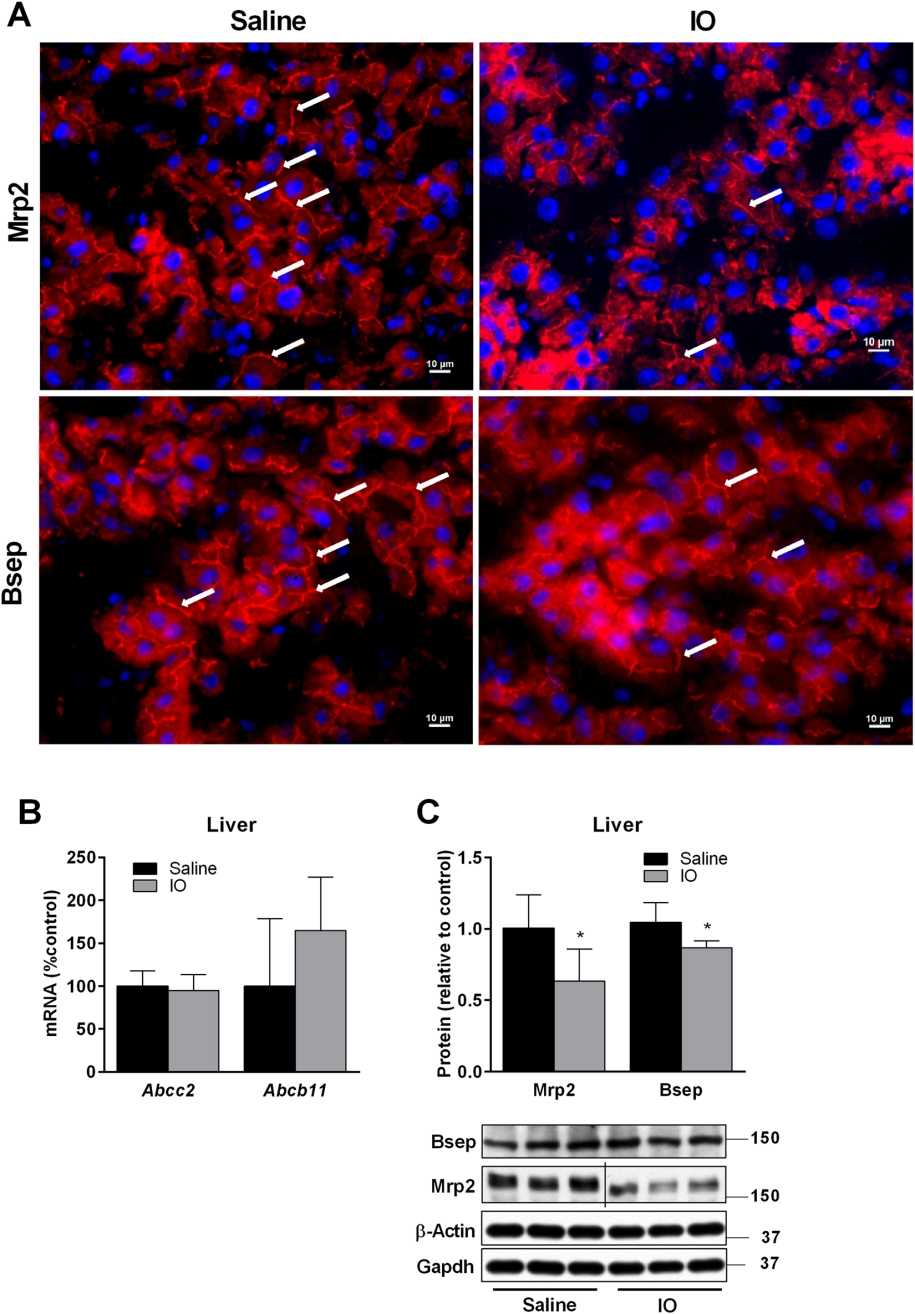
IO reduced posttranscriptionally the expression of Mrp2 and Bsep transporting proteins. (**A**) Immunohistochemical staining was used to detect Mrp2 and Bsep expression (red, white arrows) in the liver of saline and IO treated rats. Representative images of random fields are shown. Nuclei staining in blue (DAPI). Scale bar 10 μm. Isolated mRNA (**B**) and proteins (**C**) from liver tissue were used for evaluation of gene expression and protein levels. The expression of these genes was analysed by real-time RT-PCR system and proteins by western blot immunodetection. Values are mean ± SD (n = 6 in each group). *p < 0.05, **p < 0.01, ***p < 0.001 iron-treated vs. saline-treated rats.

In order to reveal the mechanisms responsible for changes in BA biliary kinetics, we analysed liver expression of transporting proteins and enzymes with crucial functions in cholesterol and BA uptake, secretion, and metabolism. Evaluating key genes in BA and cholesterol synthesis, IO significantly reduced mRNA expression of *Cyp7a*1 and significantly increased gene expression of 3-hydroxy-3-methyl-glutaryl-coenzyme A reductase (*Hmgcr*) (Fig. 4A). Of all apical and basolateral transporters, IO significantly increased mRNA expression of *Abcb1a/1b*, *Abcc3* and *Abcb4*, respectively (Fig. 4B). These transcriptional changes were followed by proportional changes in encoded proteins. We detected downregulation of Cyp7a1 (Fig. 4C) and up-regulation of Hmgcr, the rate-limiting enzyme for cholesterol synthesis (Fig. 4C), Mdr1 (Fig. 4D), the major apical transporter for biliary excretion lipophilic drugs, and Mrp3/Mrp4 (Fig. 4D), the basolateral efflux transporters for conjugated anionic compounds such as bilirubin glucuronides and BA. However, IO also down-regulated liver protein levels of Cyp8b1 enzyme for BA synthesis (Fig. 4C), Ntcp, an essential protein for uptake of BA from portal blood to hepatocytes (Fig. 4D). Increased expressions of Abcg8, an apical efflux transporter for cholesterol from liver to canaliculus, was not followed by a corresponding change in Abcg5, which may imply that the function of this heterodimer was not increased as also suggested by unchanged cholesterol biliary secretion (Fig. 3C). Previous studies described that Bsep, the rate limiting transporter for biliary BA secretion, and Mrp2, the rate-limiting transporter for anionic compounds including conjugates of BA and bilirubin, may be regulated post-transcriptionally by increased retrieval and degradation from the canalicular membrane^22^. Therefore, we performed immunohistochemical analysis in order to evaluate localization and expression of both proteins. Immunohistochemical staining in the liver showed strong Mrp2 expression in the canalicular membrane of hepatocytes in the control rats (Fig. 5A) as described in our previous paper^23^. On the contrary, Mrp2 expression was reduced in the IO rats (Fig. 5A). Similarly, Bsep expression was also detected in the canalicular membrane of hepatocytes in the control animals (Fig. 5A). However, Bsep staining was substantially weaker in the IO rats (Fig. 5A). Gene expressions of both proteins were not changed by IO (Fig. 5B), while western blot analysis confirmed downregulation of both, Bsep and Mrp2 at protein levels (Fig. 5C). These data indicate significant posttranscriptional down-regulation of Bsep and Mrp2 by IO.

### IO changes intestinal BA turnover

**Figure 6:**
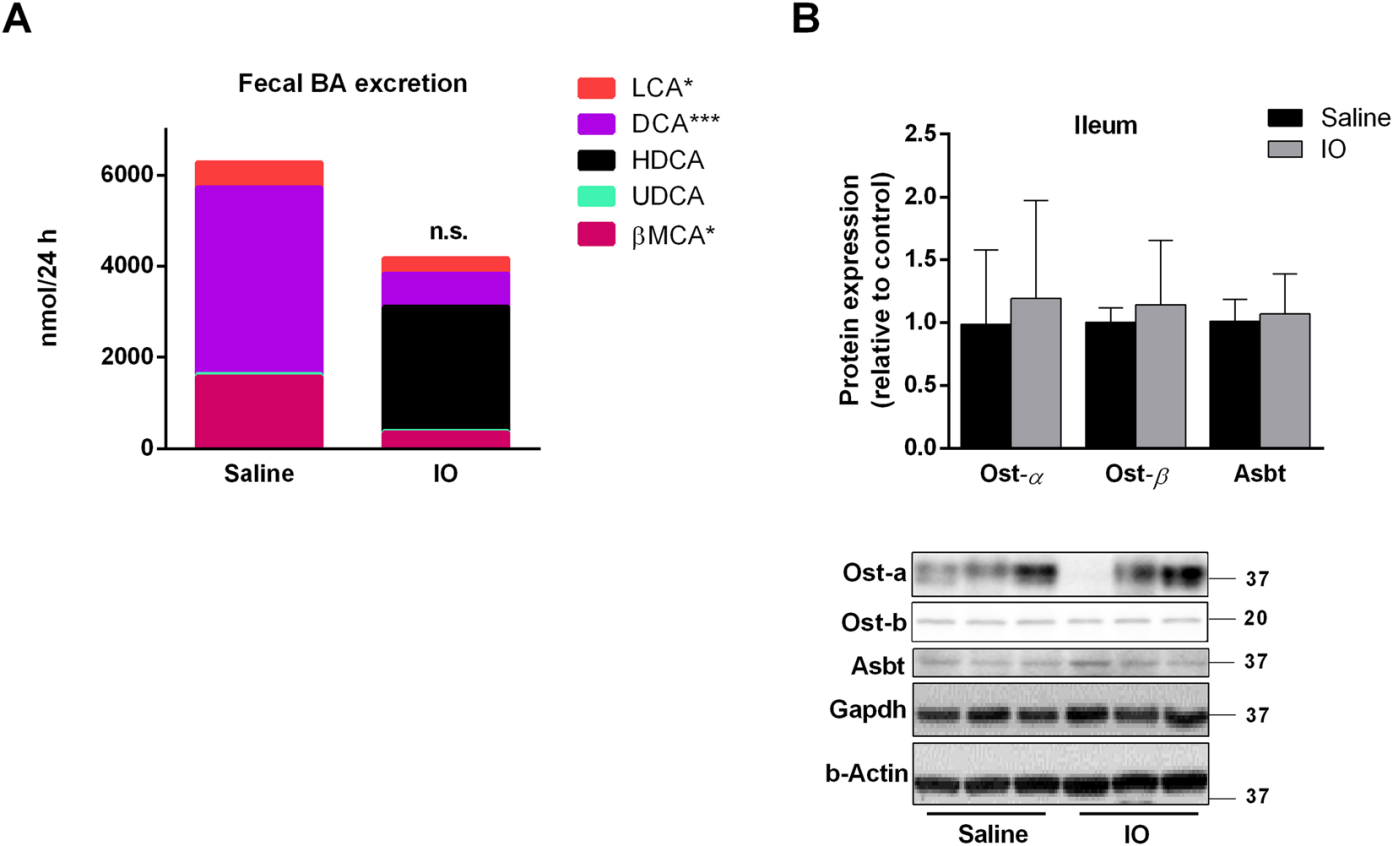
Iron overload changed intestinal BA turnover. (**A**) The BA were isolated from dried stool after 24 h collection. The concentrations of total and individual BA in bile and plasma were measured by LC-MS analysis. (**B**) Protein expression of major transporters for BA reabsorption in ileum was determined by western blot analysis and calculated to average of β-actin and Gapdh. Values are mean ± SD (n = 6 in each group). *p < 0.05, **p < 0.01, ***p < 0.001 iron-treated vs. saline-treated rats.

More than 90% of BA is reabsorbed from the ileum into portal blood, and subsequently reused by hepatocytes for secretion into the bile. In order to study intestinal turnover of BA, we analysed BA loss through faeces. BA were present in stool only in unconjugated form. In contrast to biliary secretion, the net faecal excretion of BA was highly variable between individuals; therefore, the tendency for decreased net faecal excretion of BA in the IO rats failed to reach statistical significance (Fig. 6A, Supplementary Table 3). This suggests that potential hepatic retention of BA in response to reduced biliary secretion of BA was compensated for by their reduced ileum reabsorption. Western blot analysis did not confirm significant changes in protein expression of Asbt, and Ost α/β, the major transporters for BA reabsorption at apical and basolateral membranes of ileum enterocytes, respectively (Fig. 6B). We therefore focused on faecal content of individual BA. Liquid chromatography–mass spectrometry (LC-MS) analysis revealed that faecal BA loss was reduced for the majority of BA in the IO rats, with the exception of hyodeoxycholic acid (HDCA) (Fig. 6A). HDCA was present in significant amounts in four out of six IO rats, and produced such variability in net stool BA content. HDCA was absent in the faeces of all saline-administered rats. Interestingly, concentrations of HDCA in plasma from portal bloods were 12.4 ± 7.5 μM in control and 13.5 ± 5.3 μM in IO groups, respectively, and they were not statistically different (P = 0.77). This indicates that metabolic conversion of BA by gut microbiota may modify reduced biliary secretion of BA in IO rats.

## Discussion

Alteration of cholesterol homeostasis by IO was reported by several studies, but the collected results indicate discrepancies between different models. While humans and mice^24^ with genetically determined IO such as thalassaemia or hereditary haemochromatosis develop mostly reduced cholesterol plasma concentrations, rodent models based on iron administration usually develop increased plasma^5^ or hepatic cholesterol^13^ levels. Animal models based on excessive iron administration therefore better reflect hepatic iron deposition during dysmetabolic IO syndrome, the clinical syndrome detected in about one-third of patients with NAFLD or the metabolic syndrome, and characterized by iron liver deposition and elevated plasma cholesterol^25^.

Research on molecular mechanisms explaining hypercholesterolaemia accompanying increased liver iron content has not yielded consistent results. Brunet *et al*.^5^ demonstrated reduced hepatic activities of both Hmgcr and Cyp7a1 in dietary iron loaded rats in association with increased plasma and unchanged liver cholesterol levels, and reduced biliary excretion of BA and cholesterol. Reduced gene expression of *Cyp7a1* together with increased plasma cholesterol concentrations were also detected in Hfe^−/−^ DBA/2 mice but not in Hfe^−/−^ C57BL/6 mice^12^. In another study, dietary IO mice showed positive correlation between hepatic iron content and both mRNA expression of *Hmgcr* and hepatic cholesterol content, while no relationship was seen with Cyp7a1 or with plasma cholesterol concentration^13^. Indeed, the results of this study demonstrate important new information that excessive IO may even lead to a harmful combination of Cyp7a1 downregulation coupled with marked induction of Hmgcr. We speculate that discrepancies reported by available studies regarding IO-induced changes in both HMG-CoA reductase and Cyp7a1 are related to underlying pathology and different degree and localization of iron liver accumulation.

Liver Hmgcr is regulated by SREBP-2 transcription factor in response to reduced tissue cholesterol content^26^. Thus, unchanged liver cholesterol concentrations in our IO rats suggest another factor activating SREBP-2. Indeed, recently it has been described that SREBP-2 may be induced by reactive oxygen species (ROS)^27^. ROS production typically occurs during IO^6^. Reduced liver GSH/GSSG ratio and induced Hmox1 expression and NF-κB p65 phosphorylation confirmed marked oxidative stress in the IO rats. We therefore suggest that induction of liver ROS–SREBP-2 pathway is responsible for Hmgcr induction in IO rats. The absence of cholesterol accumulation in the liver together with its unchanged biliary excretion suggests that increased plasma cholesterol concentrations are related to its increased output from the liver to the bloodstream in response to increased synthesis by induced Hmgcr, and reduced metabolism to BA due to reduced Cyp7a1. The finding of induced Hmgcr also indicates potential therapeutic strategy by statins, the Hmgcr-blocking drugs which indeed showed beneficial effects in NASH, a syndrome associated with increased incidence of liver iron deposition^28^.

The reduction of *Cyp7a1* gene expression in IO rats together with its recently detected induction during iron depletion^29^ suggests that iron regulates Cyp7a1 expression by a transcriptional mechanism. Our recent study excluded involvement of major pathways regulating *Cyp7a1* transcription such as nuclear receptors (e.g. FXR or PXR) or Egf15-pERK/pJNK signalling in iron depletion-mediated induction of Cyp7a1^29^. On the other hand, Liang *et al*.^11^ recently discovered that modulation of *Cyp7a1* mRNA by iron is executed by iron-regulating proteins IRP1 and IRP2 in mice. In general, when cells are iron-deficient, IRPs bind to iron-responsive elements (IREs) in untranslated regions (UTRs) of target mRNAs such as divalent metal transporter 1 and transferrin receptor 1, and increase their expression by stabilizing the mRNAs, while IRPs binding to UTRs of ferritin or ferroportin 1 blocks the translation of these mRNAs. When iron is in excess, IRP1 acquires a 4Fe-4S cluster and creates an aconitase, while IRP2 undergoes degradation so their binding to UTRs generally declines^11,20^. Liang *et al*.^11^ demonstrated that *Cyp7a1* has a non-canonical IRE structure in its 3′-UTR that can efficiently bind both IRP1 and IRP2 and increase transcription of this enzyme. Increased liver iron content reduces IRP1 and IRP2 and consequently reduces Cyp7a1 expression, while desferrioxamine, an iron chelator, has an inducing effect. Impairment of the IRE structure in the UTR of Cyp7a1 gene abolishes the modulating effect of iron. In the present study, reduced IRP2 expression and variable changes in liver FXR-SHP and FGF15-pJNK/pERK axes (data not shown) in IO rats imply iron-IRPs-Cyp7a1 regulation as a major mechanism of Cyp7a1 downregulation in IO rats.

Transporting proteins mediating hepatocyte uptake and biliary secretion of BA and bilirubin have not been previously studied in IO animals despite the evidence of reduced biliary BA secretion^5^. We demonstrate for the first time posttranscriptional down-regulation of Ntcp, Bsep and Mrp2 transporters for BA which resembles increased retrieval and degradation of these proteins during liver inflammation^22,30^. Data from immunohistochemistry indeed confirmed significantly reduced intensity and canalicular localization of both Bsep and Mrp2 in IO livers when compared with control rats. This pattern of regulation may correspond with significant oxidative stress induced by IO. In support, we detected upregulation of Mdr1, which is induced in the liver by binding of phosphorylated NF-κB to Mdr1 promotor^31^. These changes may, together with reduced BA synthesis, contribute to reduced biliary BA secretion in IO rats, and may modify biliary excretion of numerous compounds including drugs. Furthermore, reduced uptake of BA through reduced Ntcp, and their increased output to blood through induced Mrp4 may contribute to unchanged BA plasma concentrations and prevent intracellular BA accumulation due to impaired BA biliary excretion. Downregulation of apical Mrp2, and upregulation of basolateral Mrp3 transporters for bilirubin conjugates together with induced Hmox-1 may explain increased bilirubin plasma concentration in IO rats due to its increased synthesis and reversed transport to blood.

The impact of IO on faecal excretion of BA has been unknown to date. Despite reduced biliary BA secretion in the IO rats, the net BA output by stool remained statistically unaffected. Abst, and Osts, the major transporters for BA reuptake from the intestine, were not significantly changed by IO. In contrast, analysis of BA spectra in stool displayed marked but inter-individually variable intestinal conversion of BA into HDCA in the IO rats. A previous study showed inefficient absorption of HDCA from the intestinal tract in Wistar rats and proposed that HDCA formation might be an important mechanism for controlling the body cholesterol pools^32^. In our study, statistically unchanged concentrations of HDCA in the portal blood confirms limited capacity for HDCA intestinal reabsorption, which subsequently results in marked increase of HDCA in the stool of some IO animals. Therefore, we suggest that the marked but variable metabolism of BA into HDCA by intestinal bacteria^33^ of the IO rats was the reason for statistically unchanged net stool BA excretion in comparison with saline-administered animals. Potential changes in gut microbiome composition caused by IO must be further studied. Moreover, we have detected for the first time a reduction of liver Cyp8b1, the crucial enzyme for neutral pathway of BA synthesis, in IO rats, and analysed BA spectra in these animals. Combined downregulation of Cyp8b1 and Cyp7a1 was associated with significantly reduced content of faecal DCA, the major representative of neutral pathway of BA synthesis, in IO rats. Moreover, we have detected formation of HDCA, the metabolite of muricholic acid, a typical product of the acidic pathway of BA synthesis.

In conclusion, our data showed that IO results in a complex effect on BA homeostasis combining reduced liver BA synthesis, biliary secretion and reabsorption in the intestine, with reduced uptake to hepatocytes and increased output from hepatocytes to the bloodstream. Complex changes in transporting proteins indicate possibly dysfunctional elimination of numerous substrates including drugs. We propose that these abnormalities developed in response to oxidative stress, and IRP2 repression produced by excessive liver iron deposition. IO markedly influenced faecal excretion of BA, and our data emphasize the necessity for simultaneous evaluation of biliary BA excretion together with their intestinal processing by transporters and the gut microbiome. The interaction between iron and intestinal bacterial colonization requires further study. Finally, we further elaborate the mechanisms responsible for increased plasma cholesterol and bilirubin concentrations accompanying IO, which may serve as a potential therapeutic target.

## Methods

### Animals

Male Wistar rats (200–250 g) obtained from Velaz (Prague) were used for the experimentation. The rats were fed a standard diet under controlled environmental conditions: 12-h light-dark cycle; temperature 22 ± 1 °C with free access to food and water. The rats were randomly divided into two groups (n = 6). The control group (saline) was administrated i.p. with physiological saline (1 ml/kg) and the IO group was administrated i.p. with 8 doses of gleptoferron (iron-dextran heptonic acid complex, 100 mg/kg) every other day as described previously^18^. Excessive iron accumulation in this model induces a marked increase in hepcidin production, thus significant suppression of ferroportin 1-mediated iron intestinal absorption can be expected^19^. Administration of the 7^th^ dose was followed by placement of the rats into metabolic cages where the stool was collected for 24 h. Collected stools were dried for 72 h at room temperature and BA were isolated as described previously^34^ with slight modifications. One day after the final i.p. dose, the animals were fasted overnight and the next day they were anaesthetized with sodium pentobarbital (50 mg/kg, i.p.). The common bile duct (for bile collection) and carotid artery (for plasma collection) were cannulated. The bile was collected in pre-weighted tubes for 120 min and a blood sample was taken in the middle of this period. Thereafter, samples from portal vein were taken and the animals were sacrificed by exsanguination through carotid artery, and the livers and ilea were harvested and weighed. Tissue samples, plasma, bile, and extracted stool were snap frozen in liquid nitrogen and stored at −80 °C for future analysis. All animals received humane care in accordance with the guidelines set by the institutional Animal Use and Care Committee of Charles University, Faculty of Medicine in Hradec Kralove, Czech Republic. The protocol of the experiment was approved by the same committee (No. 18293/2016-2).

### Analytical methods

Plasma AST/ALT activities and concentrations of iron, ferritin, cholesterol and bilirubin were measured by routine laboratory methods on a Cobas Integra 800 (Roche Diagnostics, Mannheim, Germany). Biliary concentrations of phospholipids were determined by Phosphatidylcholine Assay kit (Sigma-Aldrich, St.Louis, USA). Concentrations of reduced (GSH) and oxidized (GSSG) glutathione were analysed separately using the validated HPLC method with fluorescence detection as described previously^35^. The liver concentration of cholesterol was assayed by the commercial Cholesterol Assay Kit (Cayman Chemical, Michigan, USA) and the concentration of cholesterol in bile was measured by the commercial kit Cholesterol (Erba Lachema, Brno, Czech Republic). BA concentrations in plasma, bile and stool were measured using the LC-MS method described previously^29^.

### Quantitative real time RT-PCR

Gene expression analysis by mRNA quantification was performed by reverse transcription-polymerase chain reaction (qRT-PCR) on a 7500 HT Fast Real-Time PCR System (Applied Biosystems, Foster City, USA) as previously detailed^29^. The primers used for analysis are specified in Supplementary Table S1. Glyceraldehyde 3-phosphate dehydrogenase (Gapdh) gene was used as a reference for normalizing the data (Applied Biosystems, Foster City, USA).

### Western blot

The procedure was performed as reported previously^29^. Briefly, liver lysates were prepared by homogenization in an ice-cold buffer (25 mM TRIS.HCl, pH = 7.6, 0.1% w/w TRITON-X), containing 0.5 µg/ml benzamidine, aprotinine, leptine and 10 µl/ml phosphate inhibitors (Thermo Scientific Prague, Czech Republic), and supernatant prepared by centrifugation of the lysate was separated using SDS-PAGE. Proteins were blotted to PVDF membranes, which were then blocked for 1 h with 5% non-fat dry milk in Tris-buffered saline containing 0.05% Tween 20, exposed to antibodies and chemiluminescent reagent, followed by quantification of bands on X-ray films or directly on membranes (Fusion Solo S, Vilber, France). Antibodies are described in the Supplementary Table S2. Equal loading of proteins onto the gel was confirmed by immunodetection of Gapdh and β-actin.

### Histology

The livers were collected immediately after death, fixed in 10% neutral buffered formalin, embedded in paraffin, and cut to 4–5 μm thick sections. These were stained with haematoxylin-eosin for assessment of liver morphological changes and with Prussian blue for the presence of iron. Sections were assessed by the same person using a BX-51 light microscope (Olympus) at x100 of original magnification. Ten visual fields were analysed per liver section from each animal.

### Immunohistochemistry of Mrp2 and Bsep

Five slides from each animal from each group were taken for immunohistochemical analysis. Serial cross-sections (7 μm) were cut on a cryostat and placed on gelatine-coated slides. Before antigen detection, the slides were incubated with anti-avidin and anti-biotin solutions (Vector Laboratories, USA). Thereafter, the slides were incubated with primary antibodies and after that biotinylated goat anti-rabbit secondary (Jackson ImmunoResearch, USA) (diluted 1:100 in BSA) and ExtraAvidin red fluorochrome CY3 (Sigma Chemical, USA) were used (diluted 1:300 in BSA) for the detection of either Mrp2 or Bsep. For nuclear counterstaining the blue-fluorescent DAPI nucleic acid stain (Invitrogen, Czech Republic) was used. Staining with nonimmune isotype-matched immunoglobulins assessed the specificity of the immunostaining. Primary antibodies included the following: mouse monoclonal antibody anti-Mrp2 (dilution 1:20, 1 h at RT), purchased from Enzo Life Sciences (USA), and rabbit polyclonal antibody anti-Bsep (dilution 1:50, 1 h at RT), purchased from Thermo Scientific (USA). Photo documentation and image digitizing from the microscope were performed with the Olympus AX 70, with a digital VDS Vosskühler (GmbH, Germany) with Image Analysis Software NIS (Laboratory Imaging, Czech Republic). Canalicular localization of Mrp2 was verified in our previous studies^23,29^ using the same antibodies by co-localization with another canalicular protein, Zo-1.

### Statistical analysis

Data are presented as mean ± SD. Differences between the groups were assessed by a two-tailed *t-*test assuming unequal variance. Six animals per group were analysed. Differences were considered significant at *P*-value less than 0.05. All analyses were performed using GraphPad Prism 6.0 software (San Diego, USA).

## Supplementary information

Supplementary infromation
