# Supplementary material for: Iron overload reduces synthesis and elimination of bile acids in rat liver
Source: Sci Rep. 2019 Jul 5;9:9780. doi: 10.1038/s41598-019-46150-7 (PMC6611795; doi:10.1038/s41598-019-46150-7)
Supplement: Supplementary file 1 — Supplementary infromation [file 41598_2019_46150_MOESM1_ESM.docx]

**Title page**

**Iron overload reduces synthesis and elimination of bile acids in rat liver**

Alena Prasnicka^a,d^, Hana Lastuvkova^a^, Fatemeh Alaei Faradonbeh^a^, Jolana Cermanova^a^, Milos Hroch^b^, Jaroslav Mokry^c^, Eva Dolezelova^d^, Petr Pavek^e^, Katerina Zizalova^f^, Libor Vitek^f^, Petr Nachtigal^d^, Stanislav Micuda^a^

^a^Department of Pharmacology, Charles University, Faculty of Medicine in Hradec Kralove, Hradec Kralove, Czech Republic

^b^Department of Medical Biochemistry, Charles University, Faculty of Medicine in Hradec Kralove, Hradec Kralove, Czech Republic

^c^Department of Histology and Embryology, Charles University, Faculty of Medicine in Hradec Kralove, Hradec Kralove, Czech Republic

^d^Department of Biological and Medical Sciences, Charles University, Faculty of Pharmacy in Hradec Kralove, Hradec Kralove, Czech Republic

^e^Department of Pharmacology and Toxicology, Charles University, Faculty of Pharmacy in Hradec Kralove, Hradec Kralove, Czech Republic

^f^Department of Medical Biochemistry and Laboratory Diagnostics, 1^st^ Faculty of Medicine, Charles University, Prague, Czech Republic

**Table 1** Pre-designed TaqMan® Gene Expression Assay kits (Life Technologies) used for quantitative real-time RT-PCR

| Gene symbol | Transporter/Receptor | Life Technologies cat. number |
| --- | --- | --- |
| Slc10a1 | Ntcp | Rn00566894_ml |
| Slco1b2 | Oatp1b2 | Rn00668623_m1 |
| Scarb1 | Sr-b1 | Rn0058588_m1 |
| Abcc3 | Mrp3 | Rn01452854_m1 |
| Abcc4 | Mrp4 | Rn01465702_m1 |
| Abcb11 | Bsep | Rn00582179_m1 |
| Abcc2 | Mrp2 | Rn00563231_m1 |
| Abcb1a | Mdr1a | Rn00591394_ml |
| Abcb1b | Mdr1b | Rn00561753_ml |
| Abcb4 | Mdr2 | Rn00562185_ml |
| Abcg5 |  | Rn00587092_m1 |
| Abcg8 |  | Rn00590367_m1 |
| Hmgcr |  | Rn00565598_m1 |
| Ldlr |  | Rn00598442_m1 |
| Cyp7a1 |  | Rn00564065_m1 |
| Cyp8b1 |  | Rn0144502_m1 |
| Cyp27a1 |  | Rn00710298_m1 |
| TNF-α |  | Rn99999017_m1 |
| IL-6 |  | Rn99999011_ml |
| Acta2 | α-Asma | Rn01759928_g1 |
| Tgf-β1 |  | Rn00572010_m1 |
| Hamp | Hepcidin | Rn00584987_m1 |
| Trfc |  | Rn01474701_m1 |
| Slco40a1 | Ferroportin | Rn00591187_m1 |
| Ftl | Ftl1 | Rn00821072_g1 |
| Gapdh |  | 4352338E |

**Table 2** Primary and secondary antibodies used in Western blot

| Protein | | Source | | Dilution | | Secondary antibody dilution | |
| --- | --- | --- | --- | --- | --- | --- | --- |
| Abcg5 | | Thermo Fisher (PA5-69249) | | 1:5000 | | 1:15 000 | |
| Abcg8 | | Thermo Fisher (PA5-78703) | | 1:5000 | | 1:15 000 | |
| Cyp7a1 | | Abcam (ab65596) | | 1:1000 | | 1:5000 | |
| Cyp8b1 | | Thermo Fisher (PA5-37088) | | 1:1000 | | 1:2000 | |
| Cyp27a1 | | Thermo Fisher (PA5-27946) | | 1:1000 | | 1:2000 | |
| Hmgcr | | Abcam (ab98018) | | 1:1000 | | 1:2000 | |
| Ldlr | | Thermo Fisher (PA5-20752) | | 1:1000 | | 1:1000 | |
| Sr-b1 | | Novus (NB400-104) | | 1:2000 | | 1:4000 | |
| Srebp2 | | Abcam (ab30682) | | 1:1000 | | 1:2000 | |
| Ntcp | | Thermo Fisher (PA5-80001) | | 1:10 000 | | 1:20 000 | |
| Oatp2 | | Millipore (AB3572P) | | 1:5000 | | 1:5000 | |
| Mrp3 | | Alexis (ALX801-019-C250) | | 1:1000 | | 1:2000 | |
| Mrp4 | | Abcam (ab77184) | | 1:2000 | | 1:4000 | |
| Mrp2 | | Alexis (ALX801-037-C125) | | 1:500 | | 1:1000 | |
| Bsep | | Abcam (ab71793) | | 1:1000 | | 1:2000 | |
| Mdr1 | | Signet Laboratories (8710) | | 1:1000 | | 1:3000 | |
| Mdr2 | | Thermo Fisher (PA5-78692) | | 1:3000 | | 1:10 000 | |
| Ireb1 (IRP1) | | Cell Signaling (D6S4J) | | 1:1000 | | 1:3000 | |
| Ireb2 (IRP2) | | Abcam (ab181153) | | 1:1000 | | 1:3000 | |
| NFκB p65 | | Abcam (ab16502) | | 1:1000 | | 1:2000 | |
| Hmox1 | | Sigma (H4535) | | 1:1000 | | 1:3000 | |
| Ostα | | Thermo Fisher (PA5-26837) | | 1:500 | | 1:10 000 | |
| Ostβ | | Bioss (bs2128R) | | 1:500 | | 1:10 000 | |
| Asbt | | Thermo Fisher (PA5-18990) | | 1:500 | | 1:5000 | |
| Gapdh | | GE-HealthCare (NA9340) | | 1:8000 | | 1:10 000 | |
| β-actin | | Sigma (AC-74) | | 1:2000 | | 1:4000 | |

**Table 3 Values of individual BA concentrations in plasma, BA biliary secretions and BA excretion in faeces.** Values are mean ± SD (n = 6 in each group). **p* < 0.05, ***p* < 0.01, ****p* < 0.001 iron-treated vs. saline-treated rats.

| **BA biliary secretion (μmol/g liver/120 min)** | | Saline | IO |
| --- | --- | --- | --- |
|  | TMCA | 0.6 ± 0.04 | 0.4 ± 0.06 ***** |
|  | TCA | 1.8 ± 0.23 | 1.1 ± 0.1****** |
|  | GCA | 0.2 ± 0.09 | 0.2 ± 0.06 |
|  | TCDCA | 0.2 ± 0.02 | 0.1 ± 0.01****** |
|  | TDCA | 0.1 ± 0.02 | 0.05 ± 0.01***** |
|  | CA | 0.2 ± 0.03 | 0.04 ± 0.01******* |
| Total | | 3.1 ± 0.2 | 1.8 ± 0.1******* |
|  | |  |  |
| **BA in plasma (μM)** | |  |  |
|  | αMCA | 0.96 ± 0.09 | 0.76 ± 0.11 |
|  | βMCA | 1.25 ± 0.17 | 1.10 ± 0.19 |
|  | CA | 4.36 ± 0.64 | 4.11 ± 0.3 |
|  | DCA | 0.35 ± 0.04 | 0.26 ± 0.04 |
|  | CDCA | 0.76 ± 0.1 | 0.54 ± 0.09 |
|  | HDCA | 0.98 ± 0.1 | 0.96 ± 0.14 |
|  | TαβMCA | 0.23 ± 0.02 | 0.24 ± 0.02 |
|  | TCA | 0.19 ± 0.02 | 0.35 ± 0.08 |
|  | GCA | 1.06 ± 0.21 | 1.4 ± 0.36 |
| Total | | 10.1 ± 0.8 | 9.7 ± 0.9 ns |
|  | |  |  |
| **BA excretion by faeces (nmol/24h)** | |  |  |
|  | βMCA | 1570 ± 397 | 337 ± 159 ***** |
|  | UDCA | 47 ± 11 | 31 ± 15 |
|  | HDCA | Not detected | 2730 ± 1250 |
|  | DCA | 4100 ± 633 | 729 ± 163******* |
|  | LCA | 561 ± 68.4 | 342 ± 52.3***** |
| Total | | 6280 ± 900 | 4170 ± 1400 ns |

**Full-length gels**

S - saline-treated rats IO - iron-treated rats

**IRP1**

**
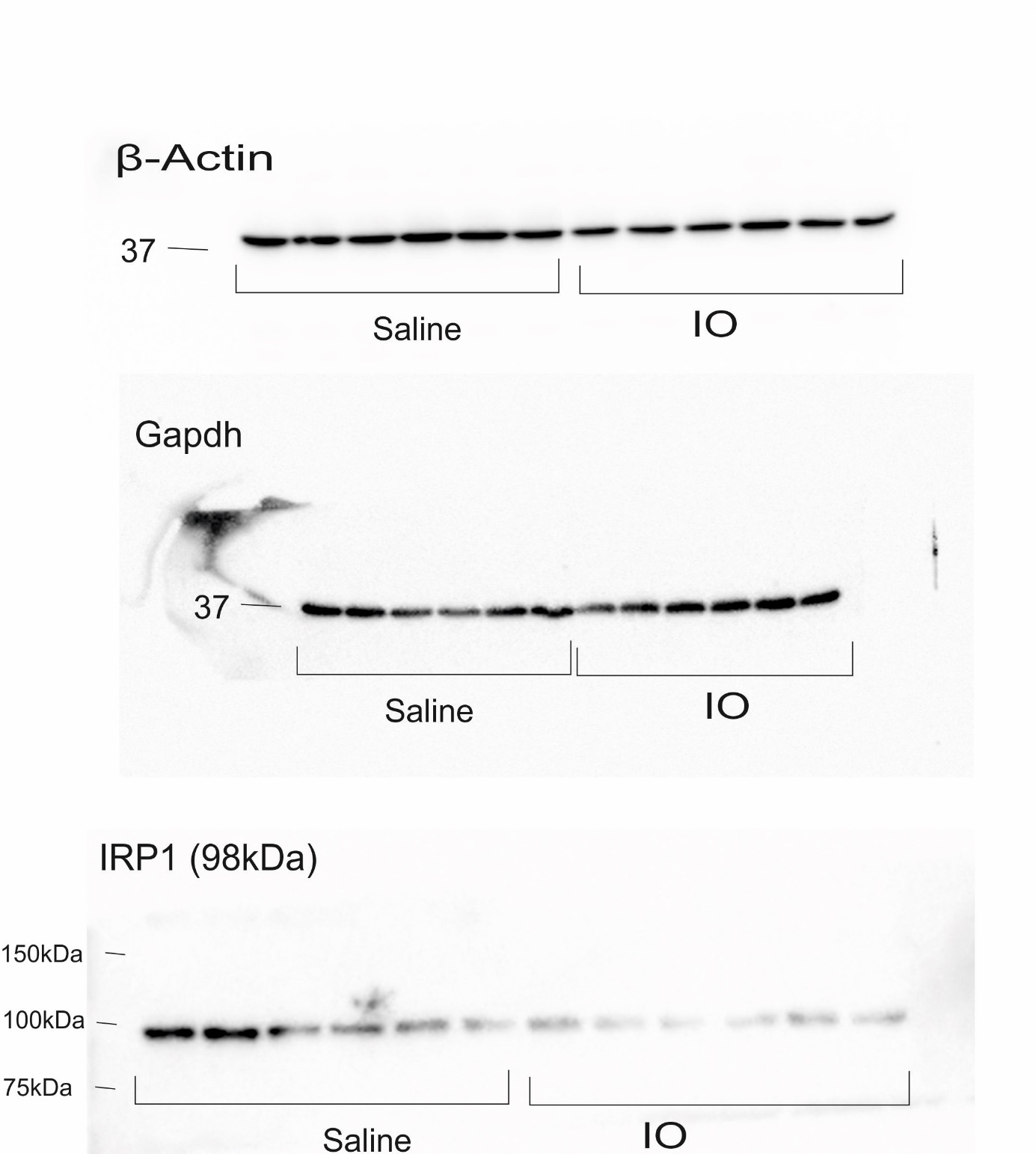
**

**IRP2**

**
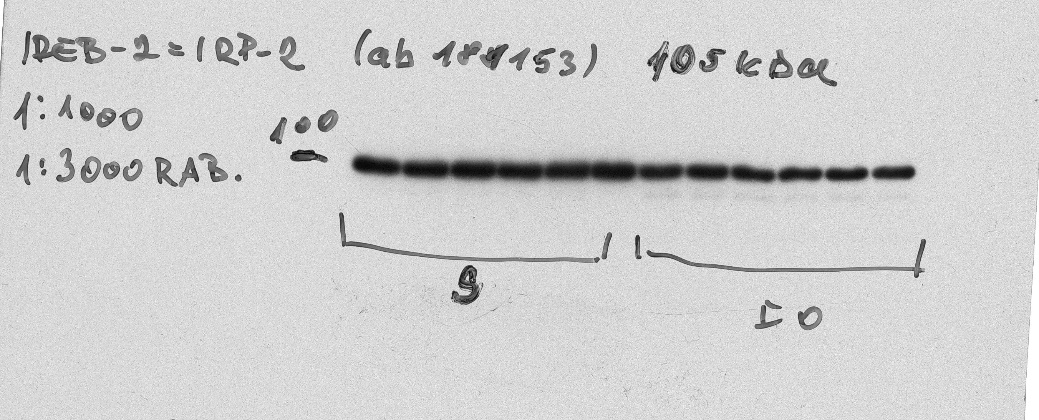
**

**β-Actin**

**
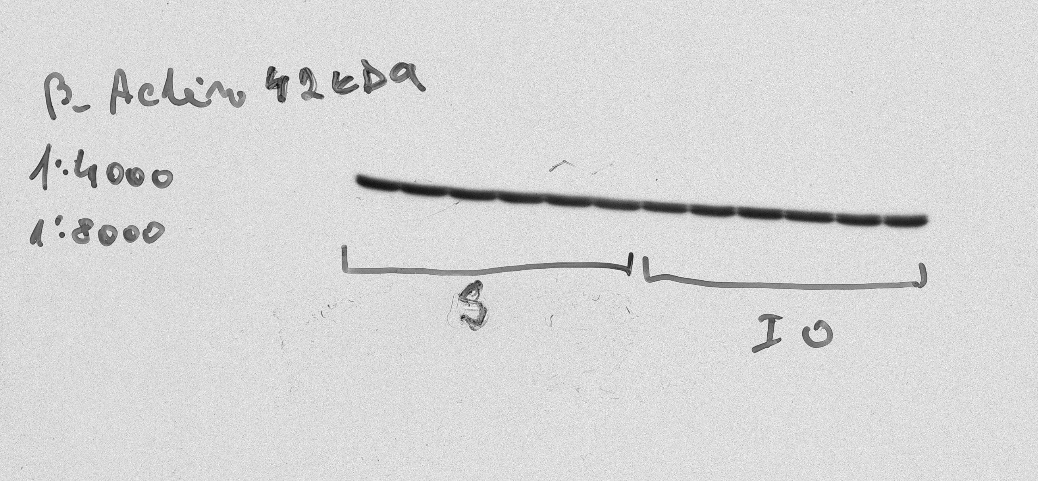
**

**Hmox1**

**
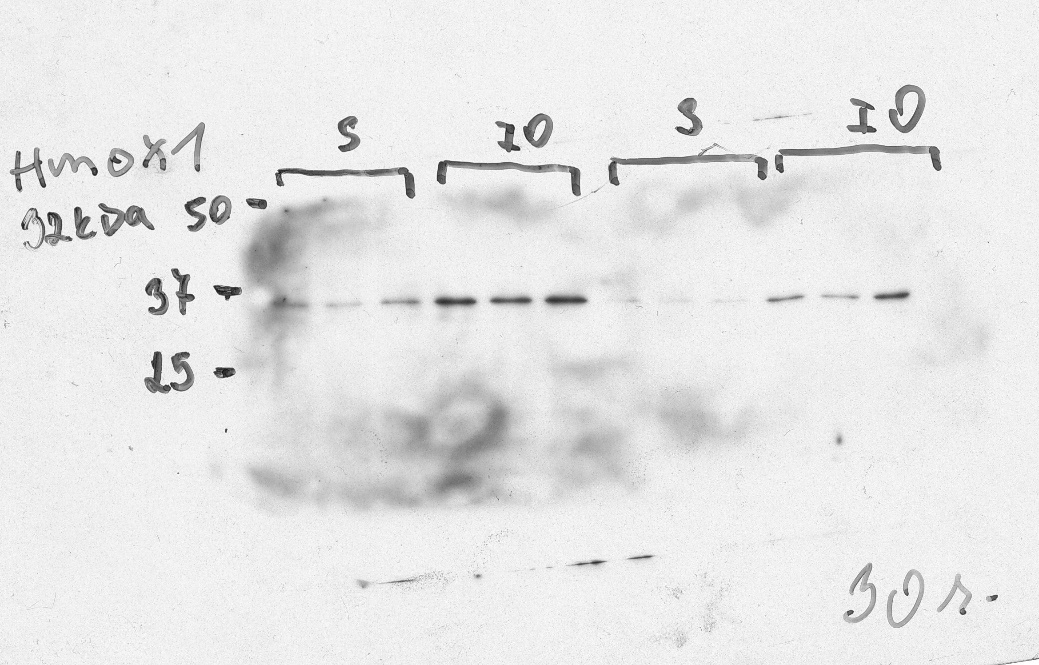
**

**NF-κB p65**

**
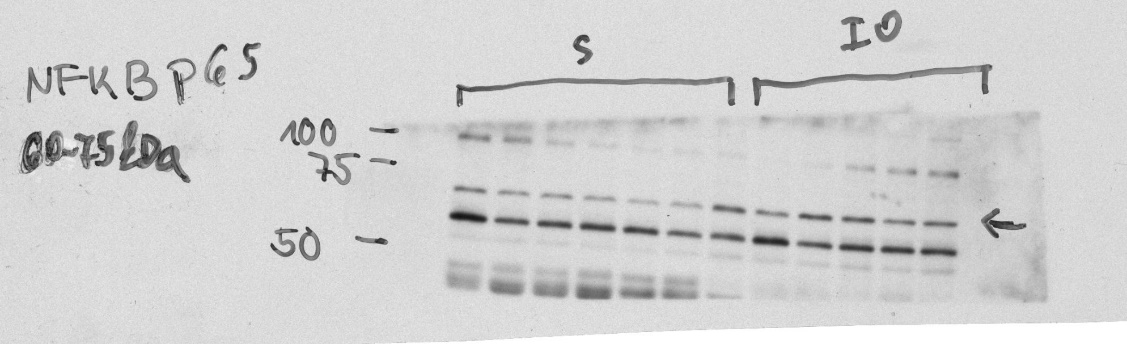
**

**Gapdh**

**
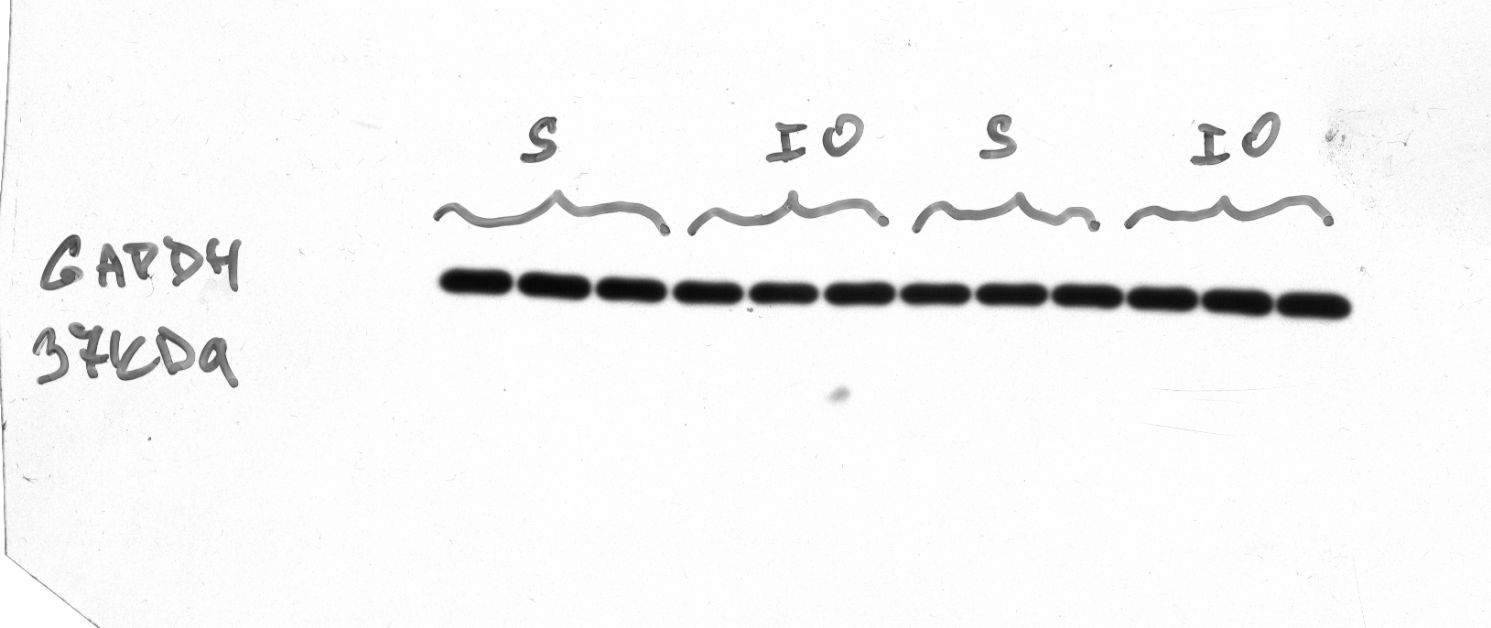
**

**β-Actin**

**
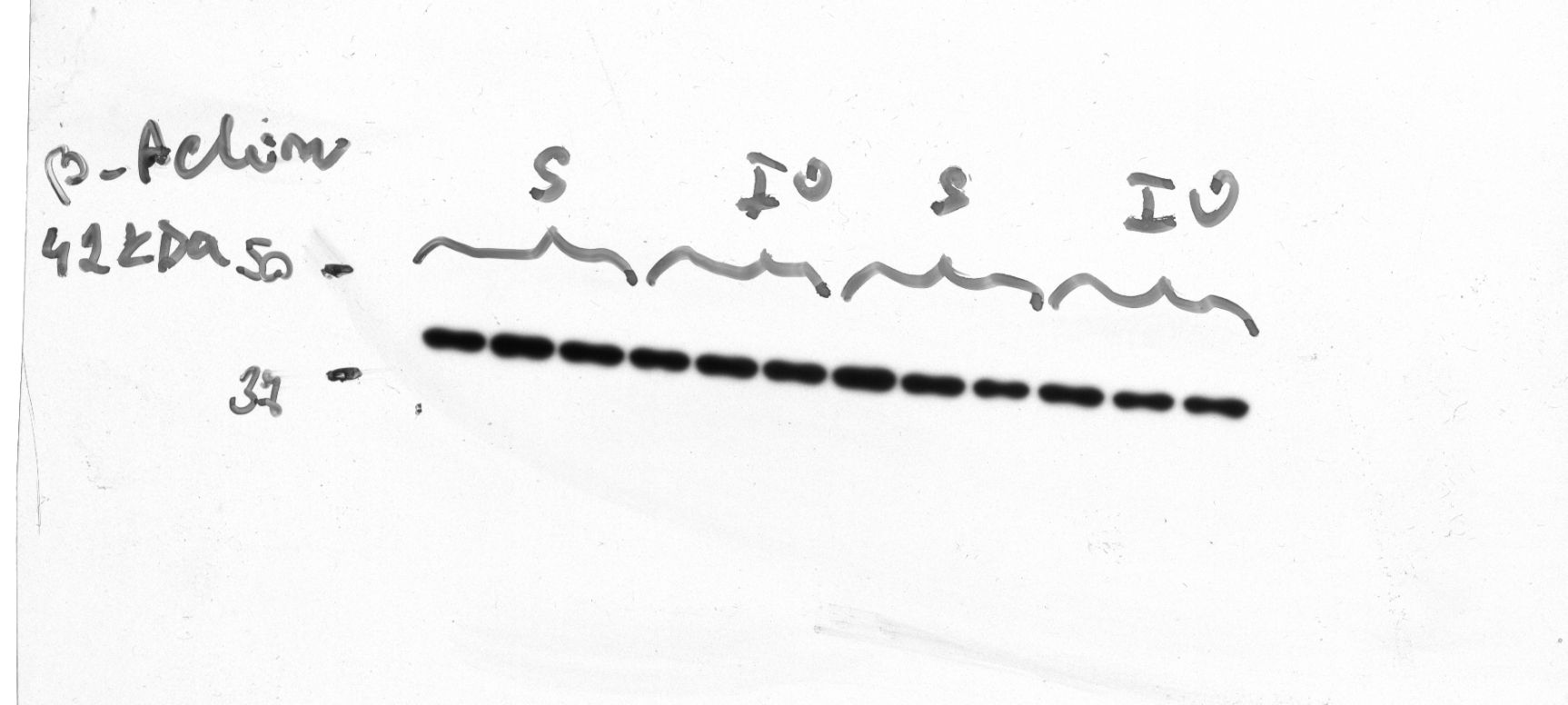
**

**Ldlr**

**
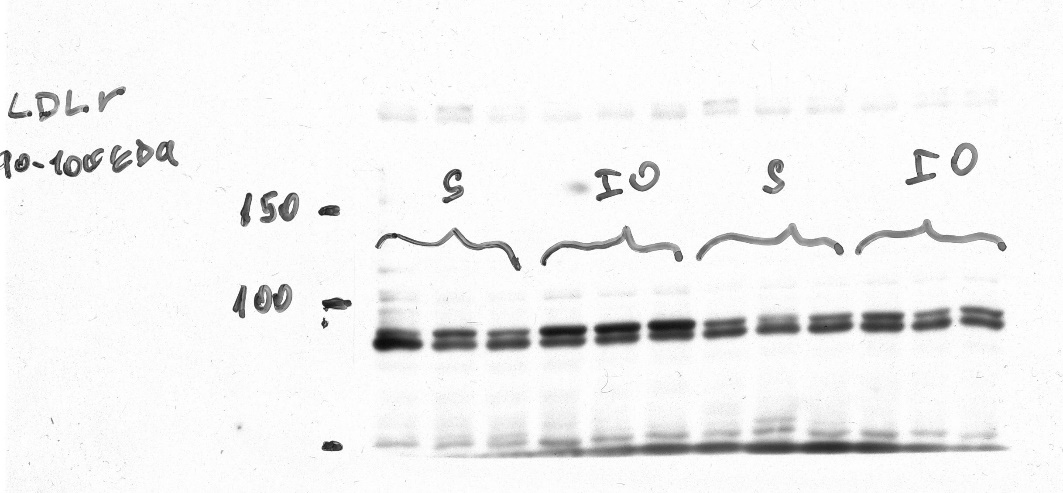
**

**Sr-b1**

**
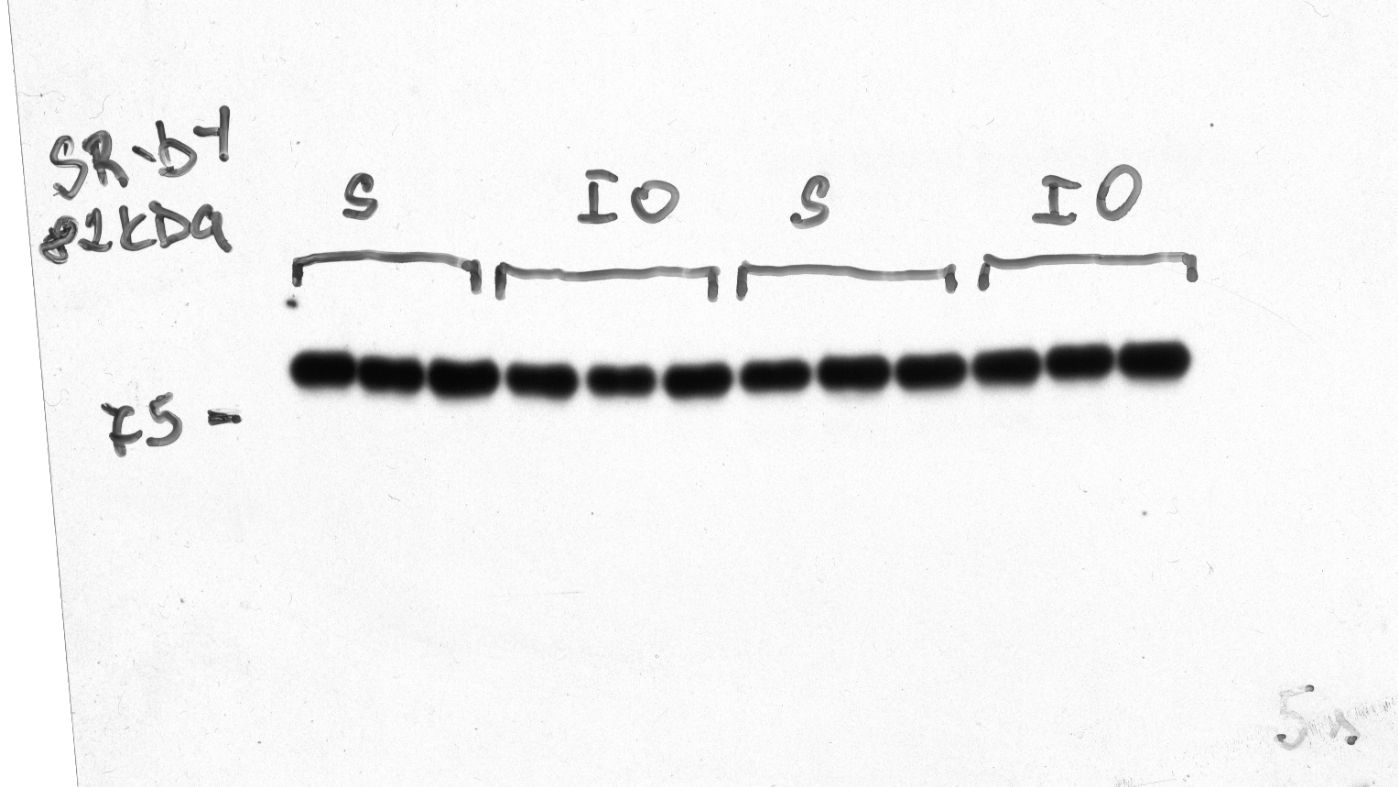
**

**Hmgcr**

**
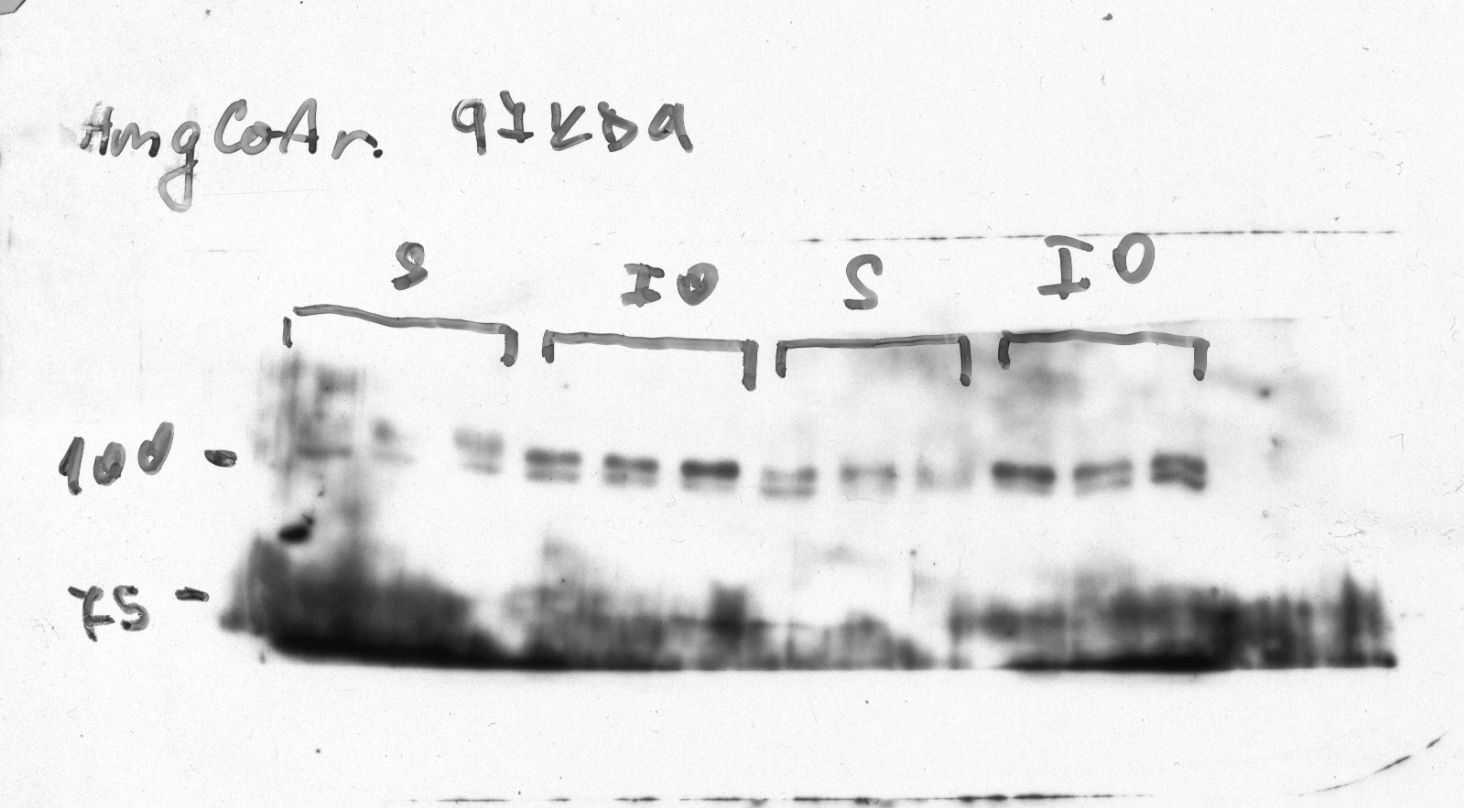
**

**Abcg5**

**
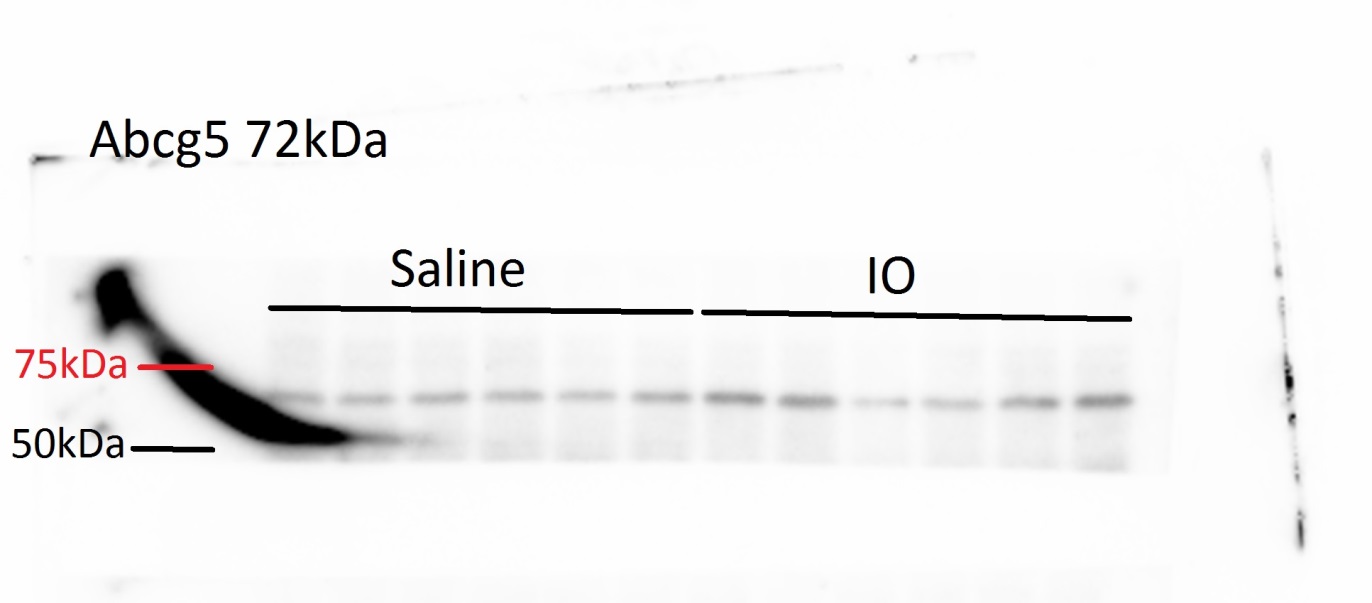
**

**Abcg8**

**
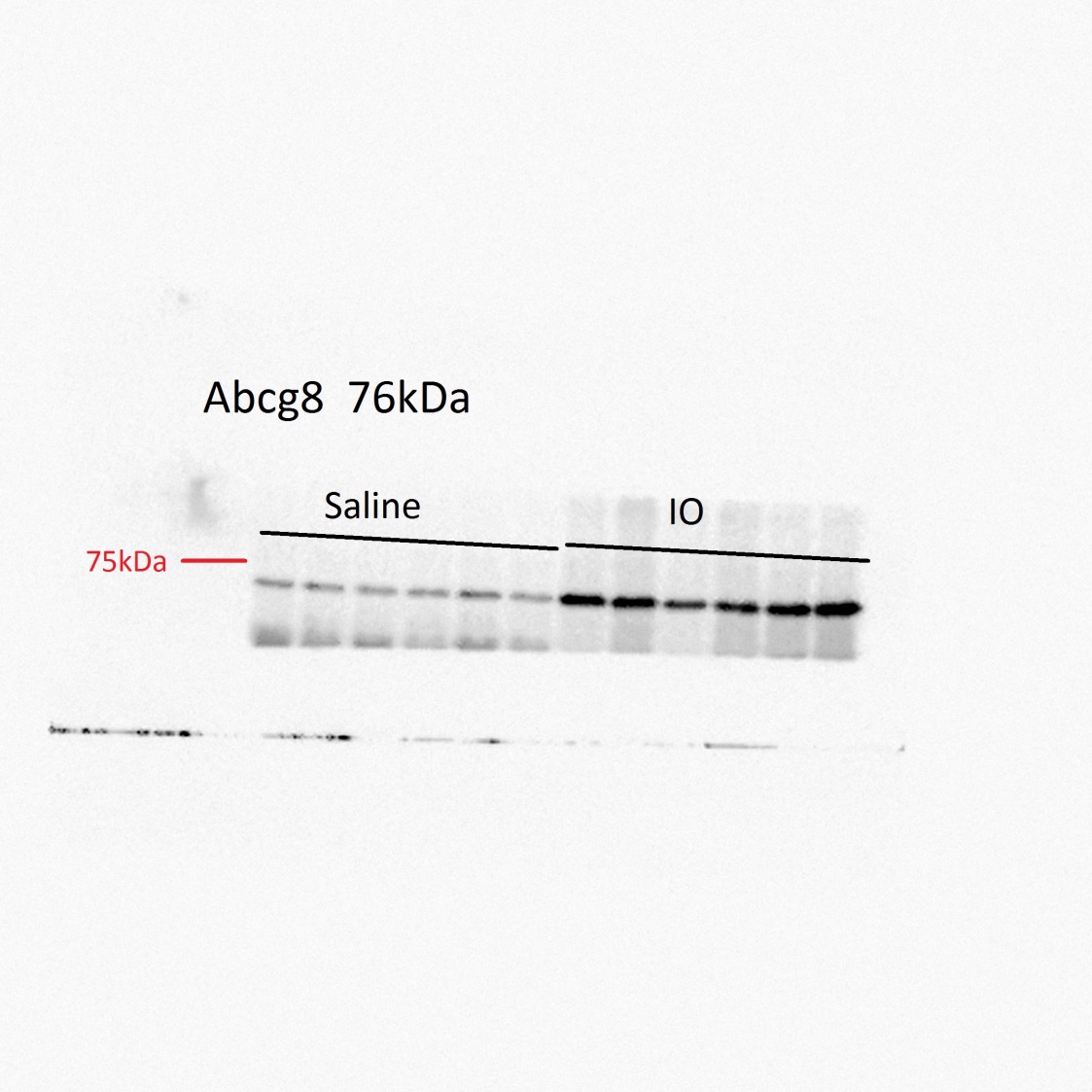
**

**Srbp2**

**
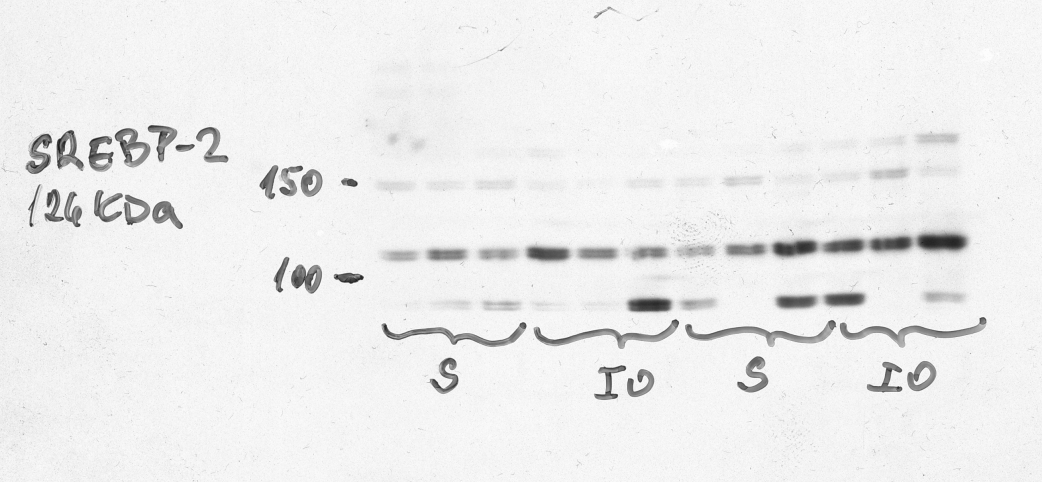
**

**Cyp7a1**

**
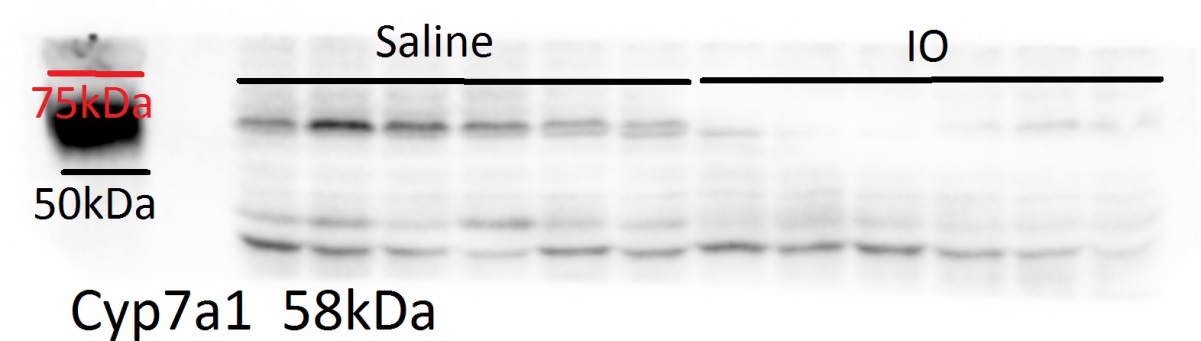
**

**Cyp8b1**

**
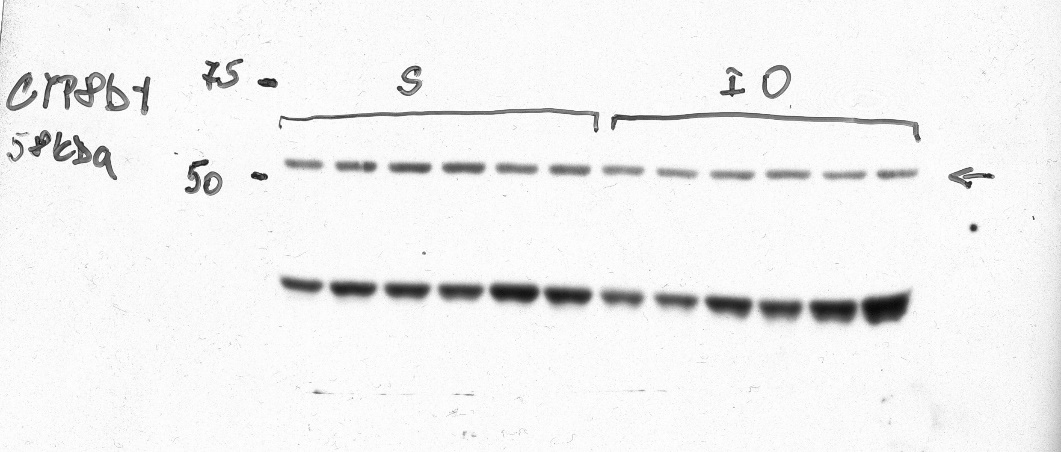
**

**Cyp27a1**

**
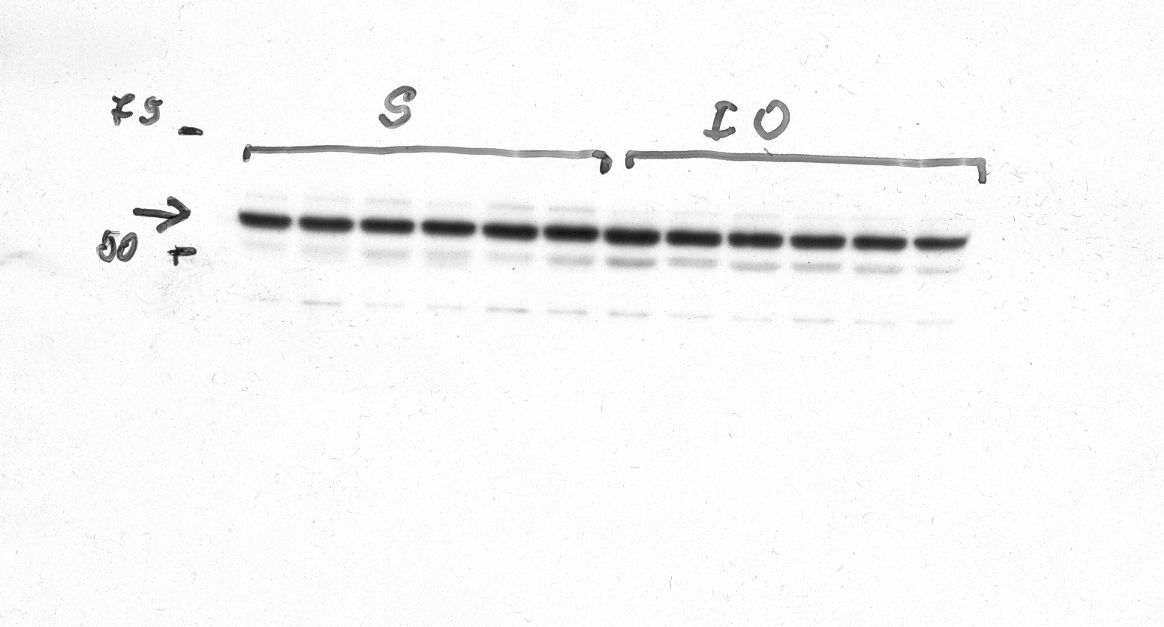
**

**Mdr1**

**
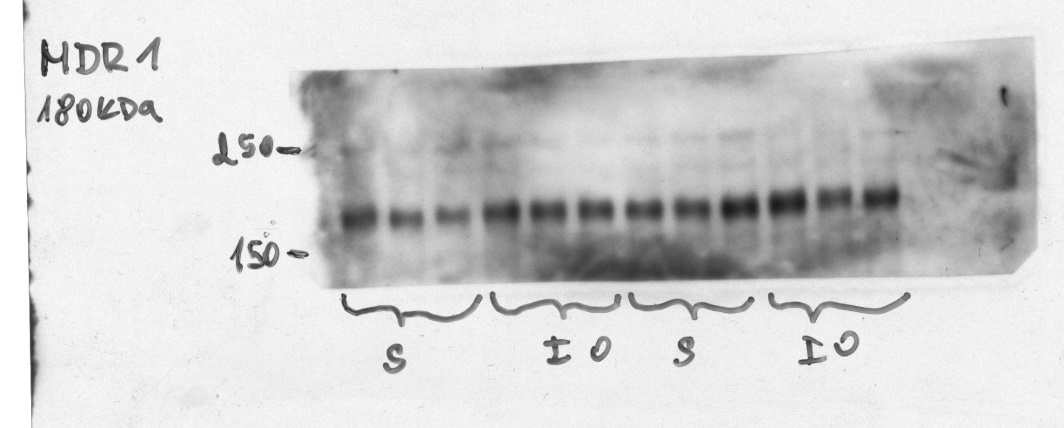
**

**Ntcp**

**
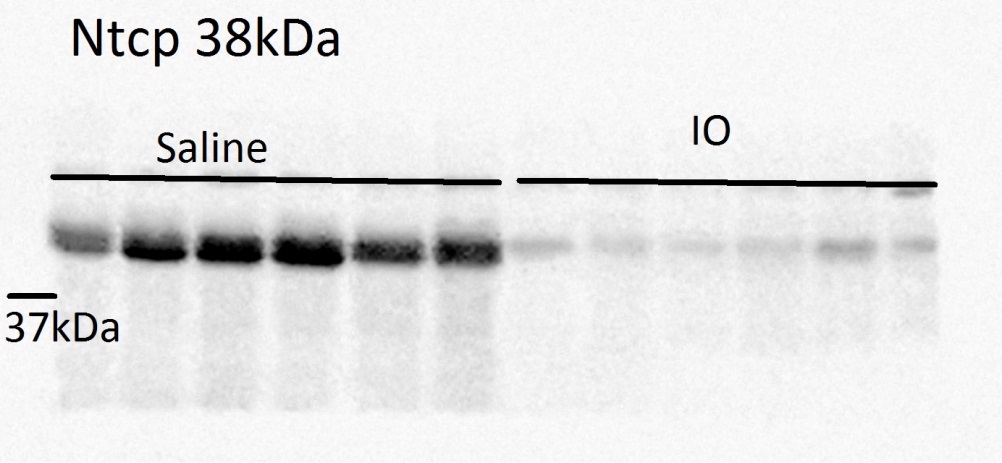
**

**Mdr2**

**
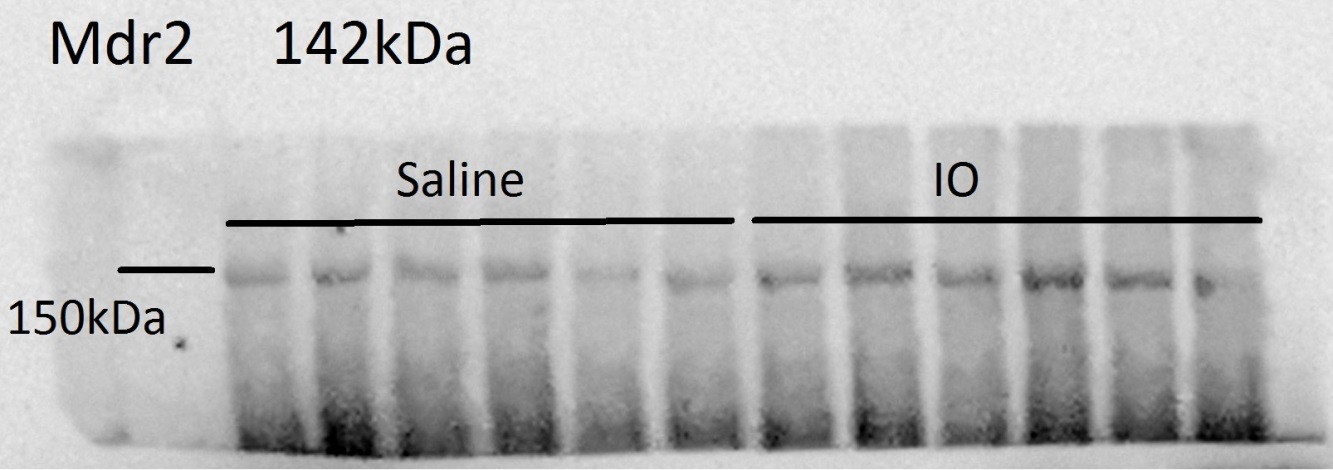
**

**Oatp2**

**
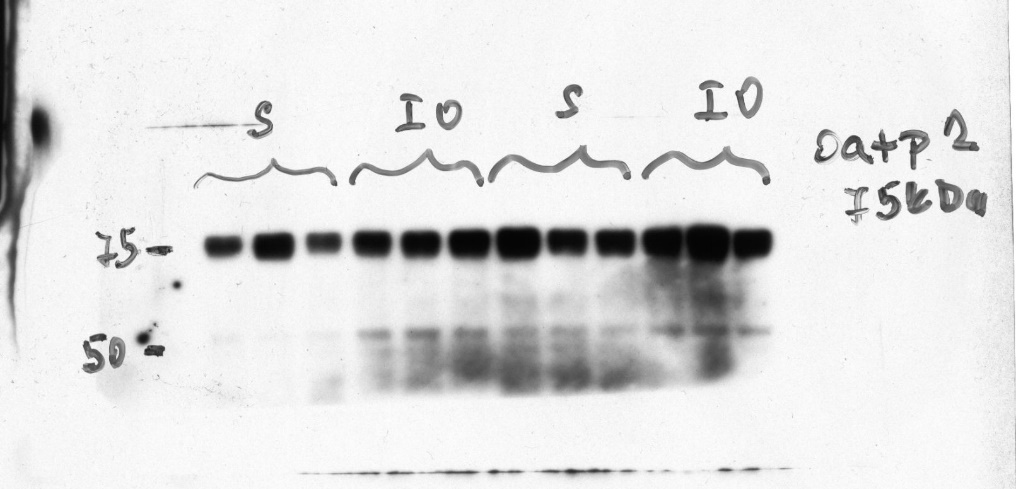
**

**Mrp3**

**
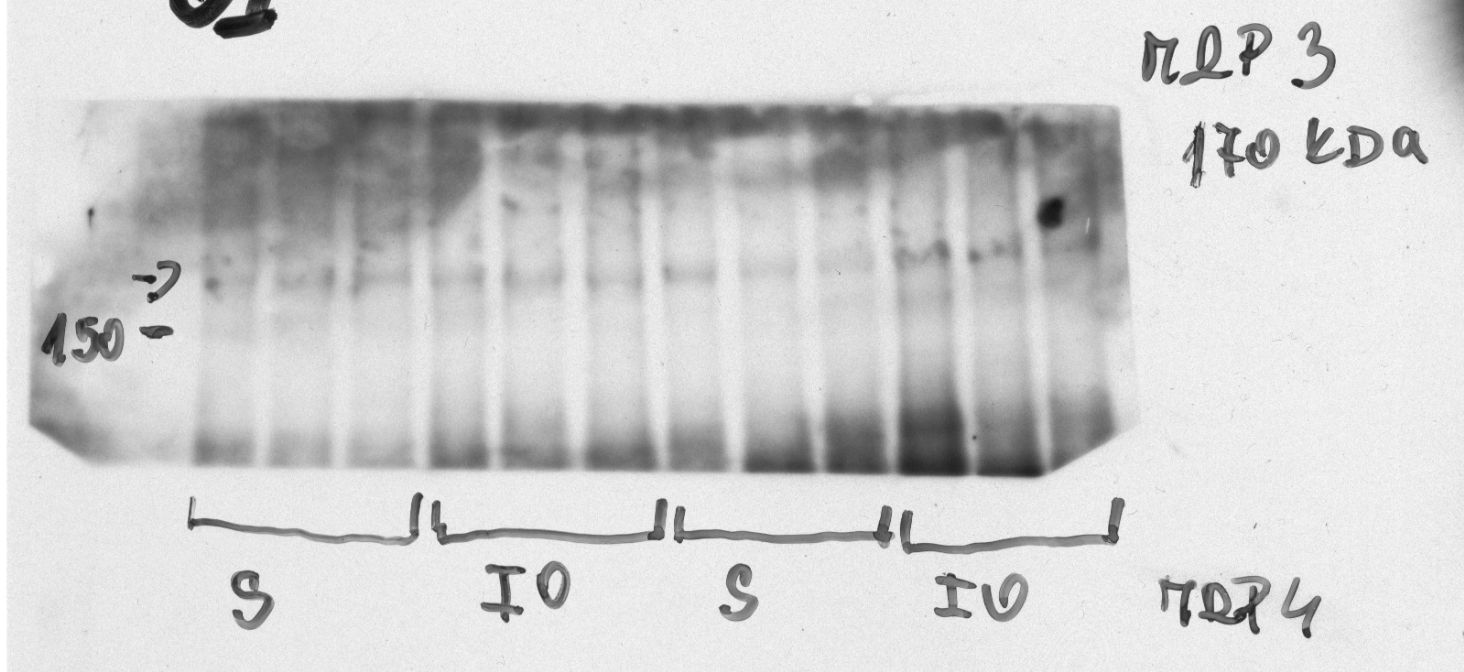
**

**Mrp4**

**
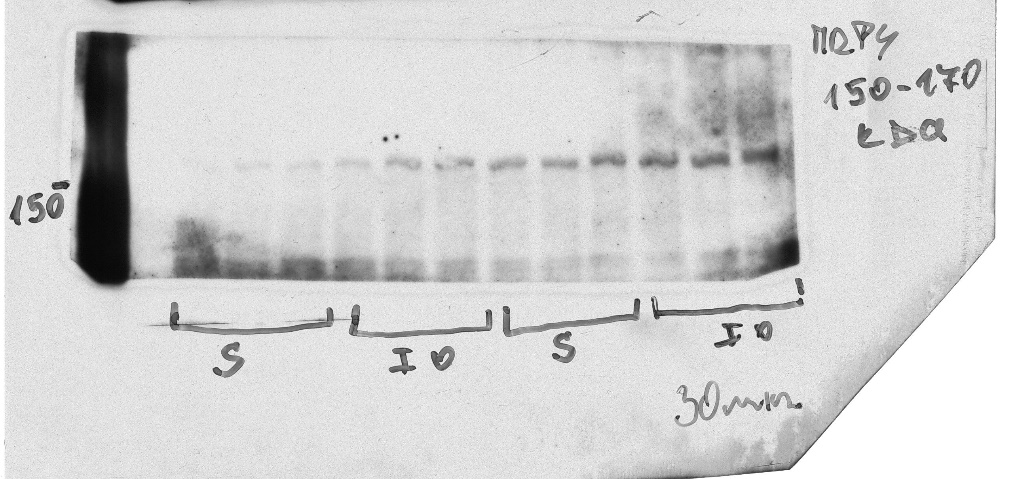
**

**Bsep**

**
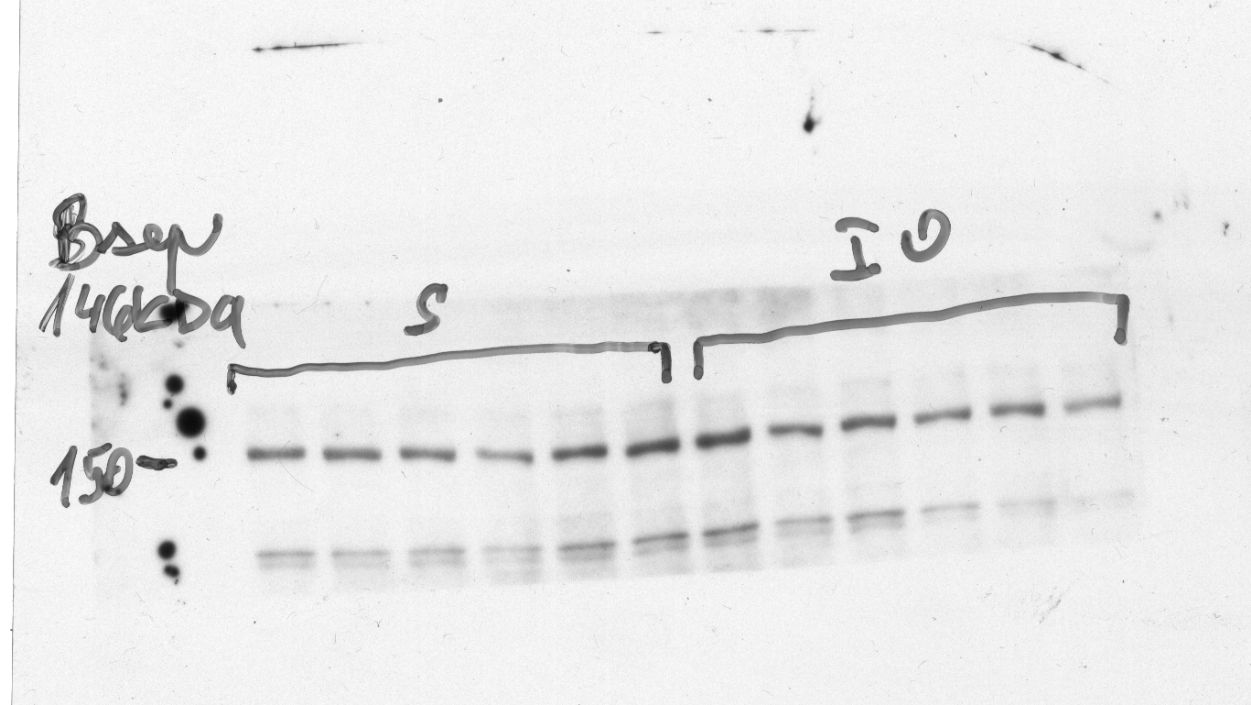
**

**Mrp2**

**
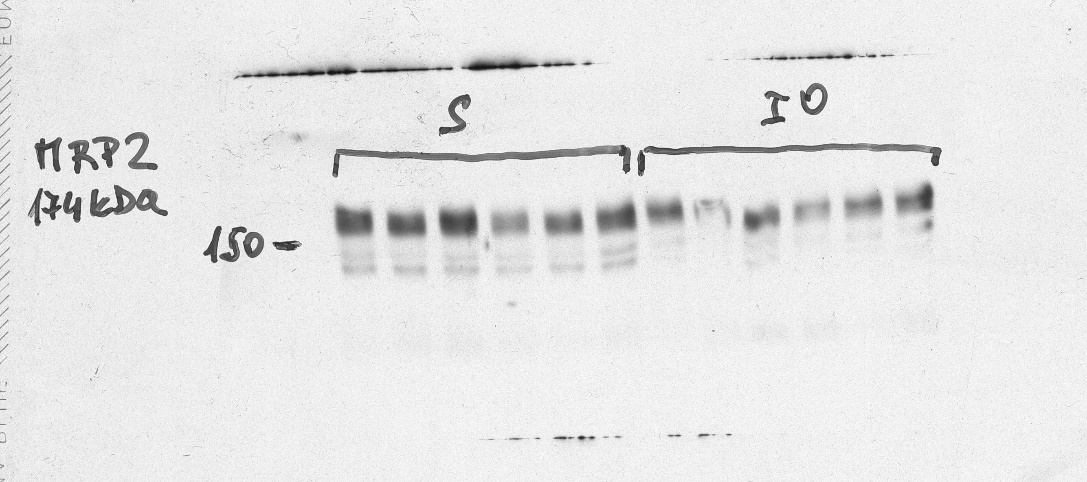
**

**Gapdh-Ileum**

**
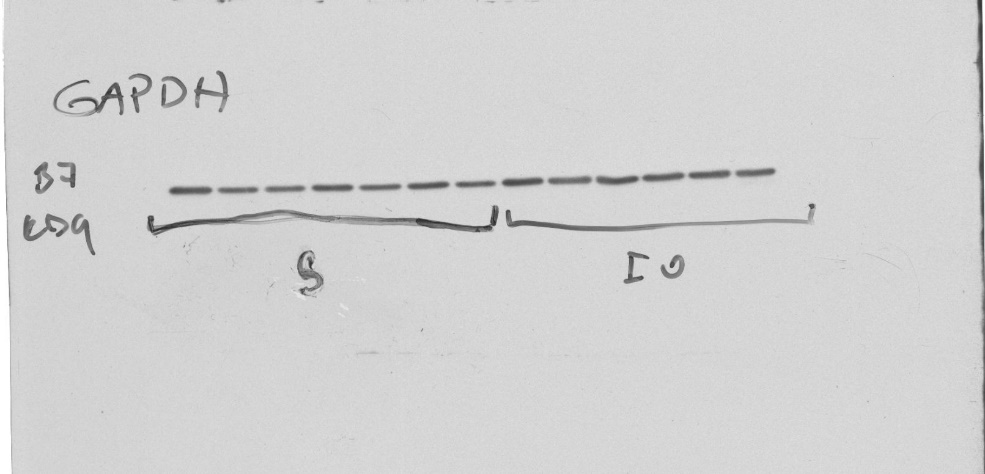
**

**β-Actin-Ileum**

**
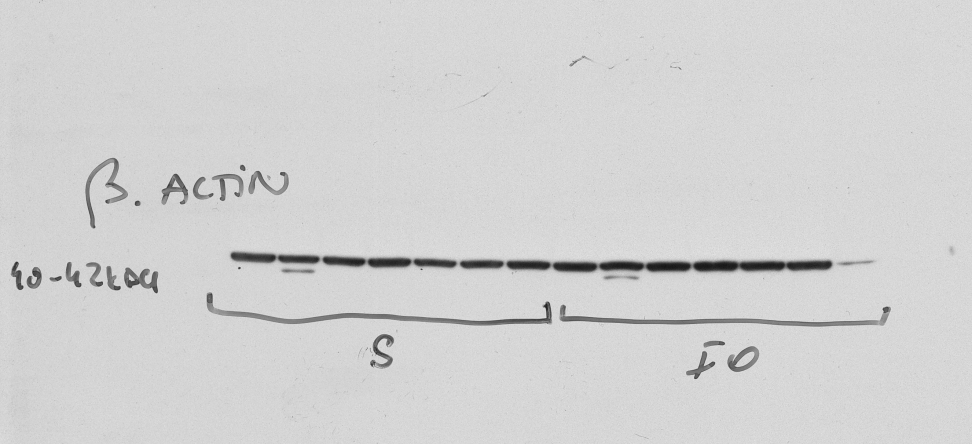
**

**Ostα**

**
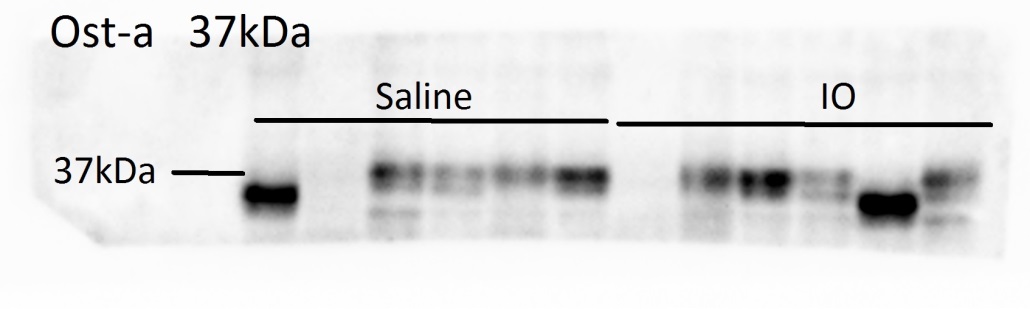
**

**Ostβ**

**
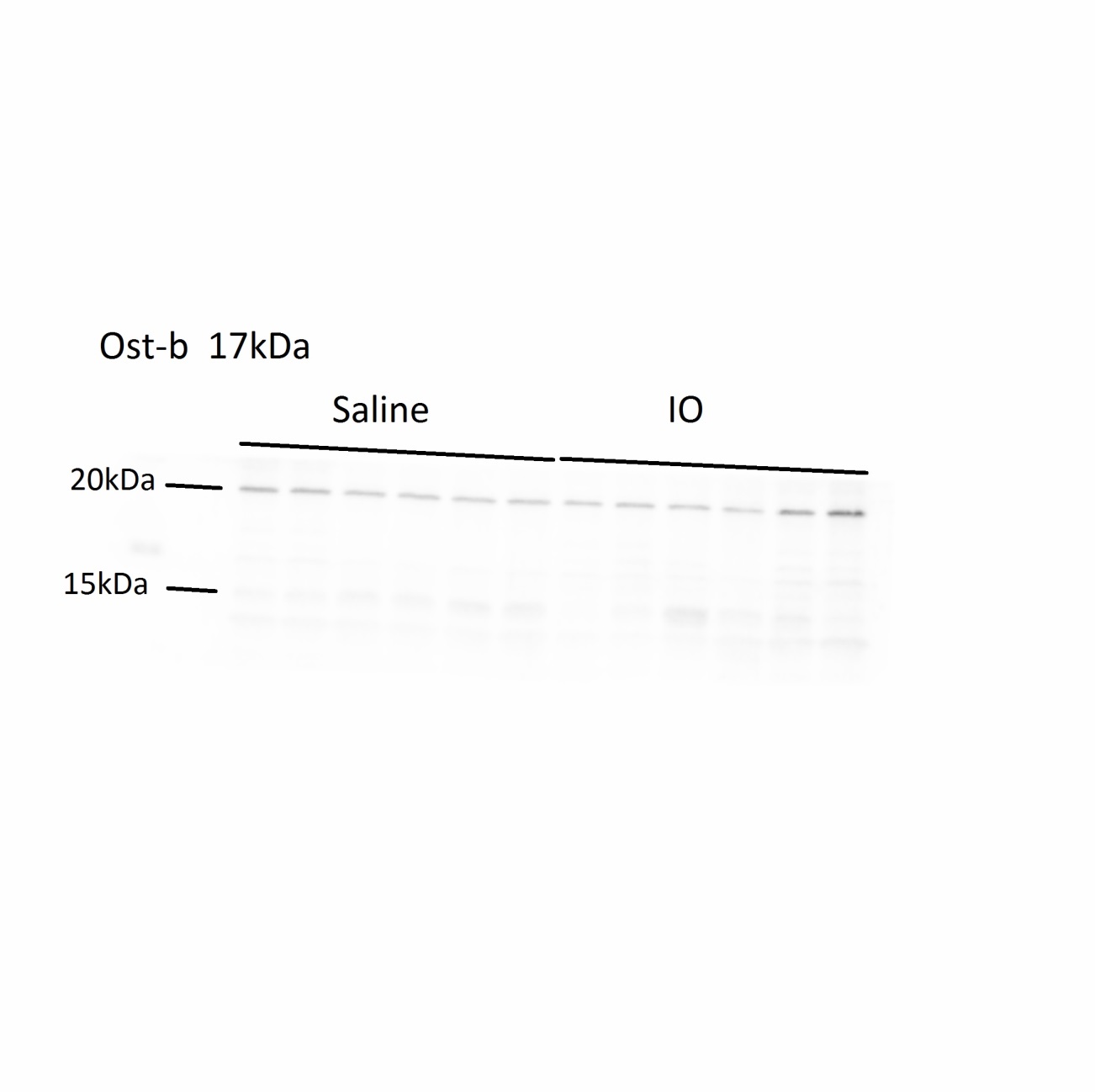
**

**Asbt**

**
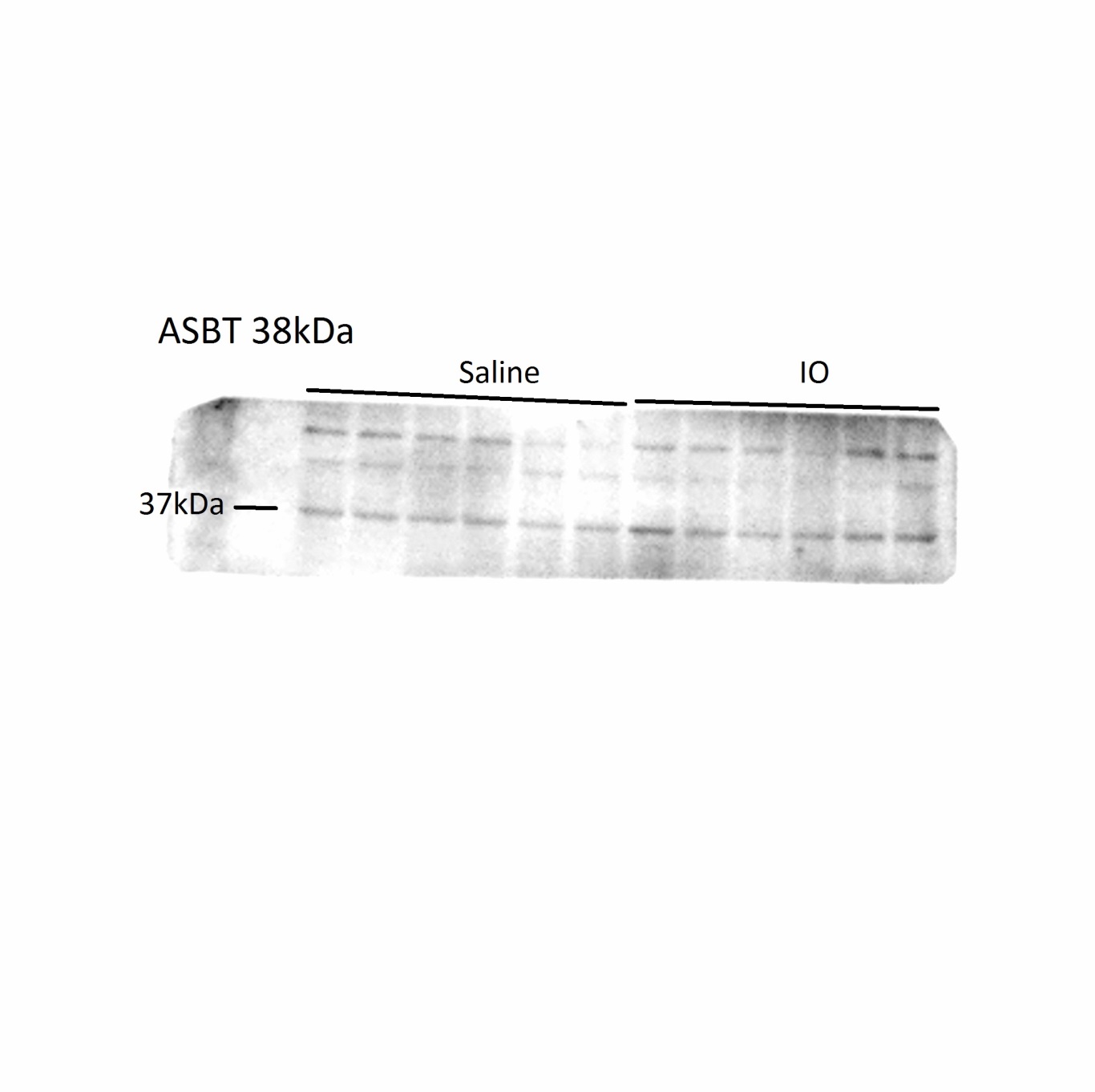
**
